# Impact of intraspecific genetic variation on interspecific competition: a theoretical case study of forage binary mixtures

**DOI:** 10.3389/fpls.2024.1356506

**Published:** 2024-09-30

**Authors:** Béatrice Wolff, Bernadette Julier, Gaëtan Louarn

**Affiliations:** INRAE UR4 URP3F, BP6, Lusignan, France

**Keywords:** multi-species grasslands, genetic diversity, individual-based model, competition, complementarity, overyielding, community stability

## Abstract

**Introduction:**

Increasing intraspecific genetic variation (IV) has been identified as a potential factor to improve productivity and stabilise botanical composition in plant communities. In grasslands systems, this could offer a lever to manage uncertainties of production and variability in the harvested species balance. However, little is known about the conditions to favour IV impact and the mechanisms at play.

**Methods:**

The dependency of IV impact on traits holding it and environmental stressors were analysed using a spatially-explicit individual-based model (IBM) of grassland communities. Sixty-three binary mixtures were defined to reflect a gradient of functional divergence between species regarding light and nitrogen (N) acquisition. The growth and dynamics of these communities were simulated for one year with three possible IV levels under two environments contrasting in terms of soil N fertility.

**Results and discussion:**

The model predicted a positive impact of moderate and high IV levels on maintaining the species balance over time, but no marked effects on mixture productivity. This stabilising effect increased at higher IV levels and under low soil N fertility. It also tended to be more pronounced in communities with intermediate functional divergence offering a significant overlap between light and N acquisition parameter values of both species. The major traits involved in the plant response to neighbours differed depending on the most contested resource, as indicated by the within-population selection of individuals with favourable N-related parameters under low N and light-related parameters under high N environments. The hypothesis that IV favours a complementarity of resource use between species was not supported. Rather, a greater spatial heterogeneity in competitive interactions was demonstrated, leading to a higher probability of growth and survival for individuals within the subordinate species. These results highlight the potential usefulness of IV to design forage mixtures with improved stability and resilience.

## 1 Introduction

The use of plant diversity in agriculture is a pillar of agroecology and a major lever to promote resilient and sustainable production systems (Li et al., 2014; Gaba et al., 2015). At the field scale, the benefits of growing at least two crop species together for a significant portion of their growth cycle (Vandermeer, 1989) have long been demonstrated in terms of yield, yield stability and resource use efficiency in the context of low input agriculture (for reviews, see Bedoussac et al., 2015; Justes et al., 2021). Mixing species that are complementary in terms of their temporal growth and resource use is already standard practice for forage production across Europe (Lüscher et al., 2014).

Recent work on plant mixtures in agriculture has emphasised the need to make a better use of genetic resources within crop species for these diversified systems. Genetic variability for mixing ability has been demonstrated in many species (e.g. Maamouri et al., 2017; Moutier et al., 2022) and cultivar choice has been shown to strongly affect the yields (Viguier et al., 2018; Kammoun et al., 2021) and ecosystem services (Thilakarathna et al., 2016) provided by such systems. There is now a clearer understanding of the assembly rules in simple binary mixtures, with key issues relating to the characterisation of cultivar traits which govern the relative advantages of species in terms of resource acquisition (light, water and nutrients), nutrient cycling and pest tolerance (Louarn et al., 2020; Stomph et al., 2020). Breeding strategies that seek specifically to improve mixing ability could be developed to improve cultivar performance in future crop mixtures (Litrico and Violle, 2015; Annicchiarico et al., 2019). Another possible option might be to increase the within-field genetic diversity of the cultivated species using current cultivar diversity (Borg et al., 2018; Snyder et al., 2020).

The positive effects of increased genetic diversity have long been detected in natural communities in terms of community stability (Booth and Grime, 2003; Clark et al., 2011; Bolnick et al., 2011; Violle et al., 2012; Ehlers et al., 2016; Westerband et al., 2021), primary productivity (Hughes et al., 2008; Whitlock, 2014) or ecosystem services (Crutsinger et al., 2006, 2009). Increasing intraspecific genetic variation (IV) at the plot level can be achieved by either mixing several varieties (Wolfe, 1985; Barot et al., 2017) or by using varieties with a broad genetic basis such as most forage species (e.g. synthetic varieties, Gallais, 1992; Smith, 2004). Positive IV effects on yield have been reported in agricultural systems, the effect usually being greater under biotic and abiotic stressors (Jokinen, 1991; Reiss and Drinkwater, 2018). In sown grasslands, maintaining communities of several species increases the agronomic and economic value of harvested forage. Greater stability in the relative proportions of species over time has been reported in some studies when increasing IV (e.g. Williams et al., 2003; Meilhac et al., 2019). However, other studies either found no effects (Fridley and Grime, 2010) or sometimes detrimental effects of IV on total yield and maintaining a desired balance of species (Zannone et al., 1983; Evans et al., 1995; Harris, 2001; Brummer et al., 2002).

A clearer understanding of how genetic diversity may affect both intra- and inter-specific plant interactions is needed so that genetic resources can be used more efficiently to improve forage crop mixtures. Mechanistic modelling can be employed to predict growth and species composition within simulated plant communities (Evers et al., 2019; Gaudio et al., 2022), also contributing to improved understanding of ecological processes in these communities (Brooker et al., 2015; Becker et al., 2022). Early modelling work of IV impact by Vellend (2006) supported niche theory expectations. It suggested that increasing genetic diversity within species could have a positive effect on the balance and coexistence of competing species whenever increased diversity allowed a selection within populations so as to reduce the functional similarity of species. However, this study neither tested the dependency of IV impact on environmental stressors nor could it allow one to infer the importance of certain plant traits to this impact. Furthermore, alternative hypotheses have since been proposed to explain IV effects on community functioning that invoke other processes such as spatial heterogeneity in access to resources, local adaptation or stochastic sampling effects (Bolnick et al., 2011; Ehlers et al., 2016). Individual-based models (IBMs) could help to advance the analysis of species interactions in such plant communities by bridging the concepts of community ecology with a mechanistic appraisal of plant trait effects on population and community scale processes (Zhang and DeAngelis, 2020). This approach was previously used to decipher the traits explaining the largest proportion of variation in the outcome of competition between intercrops (Barillot et al., 2014; Louarn et al., 2020; Li et al., 2021) or varietal mixtures (Blanc et al., 2021).

The objectives of this study were to use a spatially-explicit IBM (Virtual GrassLand model or VGL, Louarn and Faverjon, 2018) to assess the impact of increased IV in a range of hypothetical communities of two forage crop species grown in two contrasting environments. We designed these simulated communities to evaluate the impacts of variation in IV in several ways: i) to determine how the level of intraspecific trait variation and the degree of functional similarity of species (i.e. the mean trait divergence between species) affected the forage productivity and community stability of forage mixtures, and ii) to assess whether the type of competition (above- or below-ground) modified the responses and plant traits involved. A total of 63 plant communities were defined through a gradient of mean trait divergence, considering six traits (i.e. input parameters) affecting light and mineral nitrogen acquisition by plants. The growth and behaviour of all the communities were analysed for three IV levels and in two environments contrasting in terms of their soil N fertility.

## 2 Materials and methods

### 2.1 Overview of the model

The VGL model aims to simulate plant-plant interactions for multiple resources and to predict the effects of plant traits on competition and community dynamics in grasslands (Faverjon et al., 2019). The plant population model of VGL (i.e. L-egume; **Supplementary Figure A**) is a generic IBM, which deals with 3D shoot and root morphogenesis for contrasting plant morphotypes, as well as carbon (C), water, and nitrogen (N) exchanges with the environment for each individual in a population. It is based on the L-system formalism (Prusinkiewicz and Lindenmayer, 1990) and is coupled within VGL to two environmental models that deal with daily calculation of radiation transfer and light partitioning aboveground (Sinoquet and Bonhomme, 1992), and a soil model for daily water and mineral N balances below ground (Louarn et al., 2016). A detailed description of the model equations can be found in (Louarn and Faverjon, 2018). Its source code is freely available (https://github.com/openalea-incubator/l-egume; version 1.3, doi: 10.5281/zenodo.7111768). Only the principal features of the plant model are described below.

In brief, the model uses a daily time-step to compute the potential morphogenesis of shoots and roots as a result of the functioning of plant meristems and growing tissues. Potential morphogenesis frameworks, adapted from Faverjon et al. (2017) for shoots and Pagès et al. (2014) for roots, are used in the L-system. They define the spatial distribution and dimensional growth of plant exchange surfaces that interact with the environmental submodels to allow each individual to capture light (**Supplementary Figure A**) water and soil mineral N. Potential plant dry matter production is then determined from light interception by shoots using a radiation-use efficiency (RUE) approach (Monteith, 1977), which in turn defines the water and N requirements necessary to sustain maximum plant growth. Four compartments per plant (leaves, stems, taproot, and fine roots) are considered for the partitioning of dry matter and N. From this potential growth situation, two feedback loops are implemented in the model to account for plant plasticity and the regulation of growth and morphogenesis by the environment. On the one hand, light quality distribution is deemed to trigger local photo-morphogenetic responses that downregulate phytomer production by shoot axes (Baldissera et al., 2014) and modulate organ expansion (Gautier et al., 2000). On the other hand, the soil resources available to plant roots are compared with water and N requirements in order to scale actual plant growth. Two ratios accounting for water availability (fraction of transpirable soil water, FTSW) and the satisfaction of N demand (nitrogen nutrition index, NNI) are calculated from plant uptakes in the soil. These ratios define two levels of stress that are applied both systemically (whole plant level) and independently (multiplicative effects) in order to regulate plant growth and morphogenesis under limiting soil conditions.

### 2.2 New model developments to account for within population parameter variation

In local populations of most grassland species, as well as in forage cultivars selected by breeders (i.e. synthetic varieties; Smith, 2004), significant genetic and phenotypic variability is present (Julier et al., 2000). The previous version of the model did not take account of this aspect as it considered the plant population of a given cultivar through a unique vector of scalar parameter values (i.e. all plants from the same variety had identical parameter values).

In this new version of the model, each individual plant is assumed to have a singular vector of parameter values. Within a cultivar, parameter values are assumed to be drawn from a Gaussian distribution *N (µ, σ)* for which *µ* equals the former parameter value of the model for this population and *σ* the standard deviation of parameter distribution at the population level. Such a Gaussian distribution is consistent with the theory of quantitative genetics for quantitative traits, in which the genetic basis of a trait results from the effect of a very large number of loci (Fisher, 1918). Furthermore, possible genetic correlations between traits were considered by defining a covariance matrix *M* between plant parameters (i.e. a symmetric and positive semi-definite matrix, with its main diagonal containing parameter variances and its other elements giving the covariance between each pair of parameters) and by drawing all parameters simultaneously from a multivariate normal distribution law *N_m_* (Krzanowski, 2000).

These new features were introduced into the model through their standardised dimensionless forms using two new inputs (**Supplementary Figure A**): a scalar vector *CV* specifying the coefficient of variation (*σ/µ*) for each parameter and a positive semi-definite correlation matrix *M_c_* specifying the pairwise correlation coefficient between the different parameters, so that:


(1) $$M={\left(\mu .CV\right)}^{2}.M_{c}$$

By default, with *CV* equal to zero and *Mc* being a scalar matrix equal to 1, the new version of the model produces exactly the same outputs as the previous model version. However, it is also now possible to run simulations by introducing observed and scenario-driven *CV* and *Mc* values for all, or only a subsample, of the input plant parameters.

In order to summarise the parameter values of a given individual relative to the population to which it belongs, an average parameter score (P_score_) is provided by the model and calculated as the average of the standardised parameter values for all *n* parameters holding within-species variability:


(2) $$P_{score}=\sum_{n=1}^{n}S_{n}.\left(\frac{P_{n}-{µ}_{n}}{\sigma_{n}}\right)/n$$

Where *P_n_* represents the individual’s parameter value drawn in the multivariate normal distribution *N_m_* and *S_n_* represents a sign (1 or -1) accounting for the effect of increasing values of this parameter on resource acquisition.

### 2.3 Model parametrization

The VGL model was previously parametrized and assessed for a range of legume-based forage mixtures (Faverjon et al., 2019). A sensitivity analysis was also performed to assess the effects of plant parameters on the performance of such binary mixtures (Louarn et al., 2020). For the present study, the vector of mean parameter values (µ) was considered to be identical to that in a previous study using non-nitrogen fixing plants (i.e. “G- morphotype”, Louarn and Faverjon, 2018). This corresponded to an erect crown-forming dicotyledonous plant, close to the calibration for alfalfa calibration but unable to fix atmospheric N. Three possible levels of intraspecific genetic diversity were defined to account for within-population parameter distributions: very low (CV=0.001), moderate (CV=0.15) and high (CV=0.3) values. The moderate and high CV values were derived from a literature review summarised in **Supplementary Table A**. These two later thresholds corresponded approximately to the median and maximal values within the range of possible CV characterising within-species parameter variation. The very low *CV* value was defined so that a distribution of plant parameters values was generated, but resulted in no significant deviation on either of the model outputs compared to a null *CV*. In order to simplify the analyses, parameter distributions were deemed to be independent from each other and the *Mc* matrix was defined as a scalar matrix (i.e. all covariances equal to zero).

### 2.4 Virtual experiment: assessing the impact of within-population parameter variation on interspecific competition and community productivity

A series of 63 virtual plant communities was generated in order to analyse the impact of within-population genetic variation in contrasting situations of inter-specific plant interactions regarding resources. The term ‘species mixture’ refers below to a given community defined by two sets of mean parameter values (i.e. µ). Each virtual community was built using a reference species (corresponding to the G- morphotype described above; hereafter called Sp1 for species 1) and a second species (hereinafter called Sp2 for species 2) defined by modifying the values of a series of one to six parameters compared to Sp1. The term ‘mean trait divergence’ refers below to the difference in mean parameter values between the two species of a community (µSp1 – µSp2).

Six model parameters were selected to build plant communities with contrasting levels of competition between species for light and N acquisition. Three parameters were identified as having a marked influence on light competition (namely L_max_L, L_max_In and Phyllo_1_, standing for maximal leaf length, maximal internode length and maximal phyllochron of primary shoot axes, respectively; Louarn et al., 2020) and three parameters with a marked influence on soil mineral N competition (namely Vmax_2_, EL_max_R and PPtresh_H_, standing for the maximum rate of absorption achieved by Low Affinity Transporters, the root elongation rate at maximal apex diameter and the photoperiod threshold required to induce a reduction in plant development, respectively).

**Figure 1:**
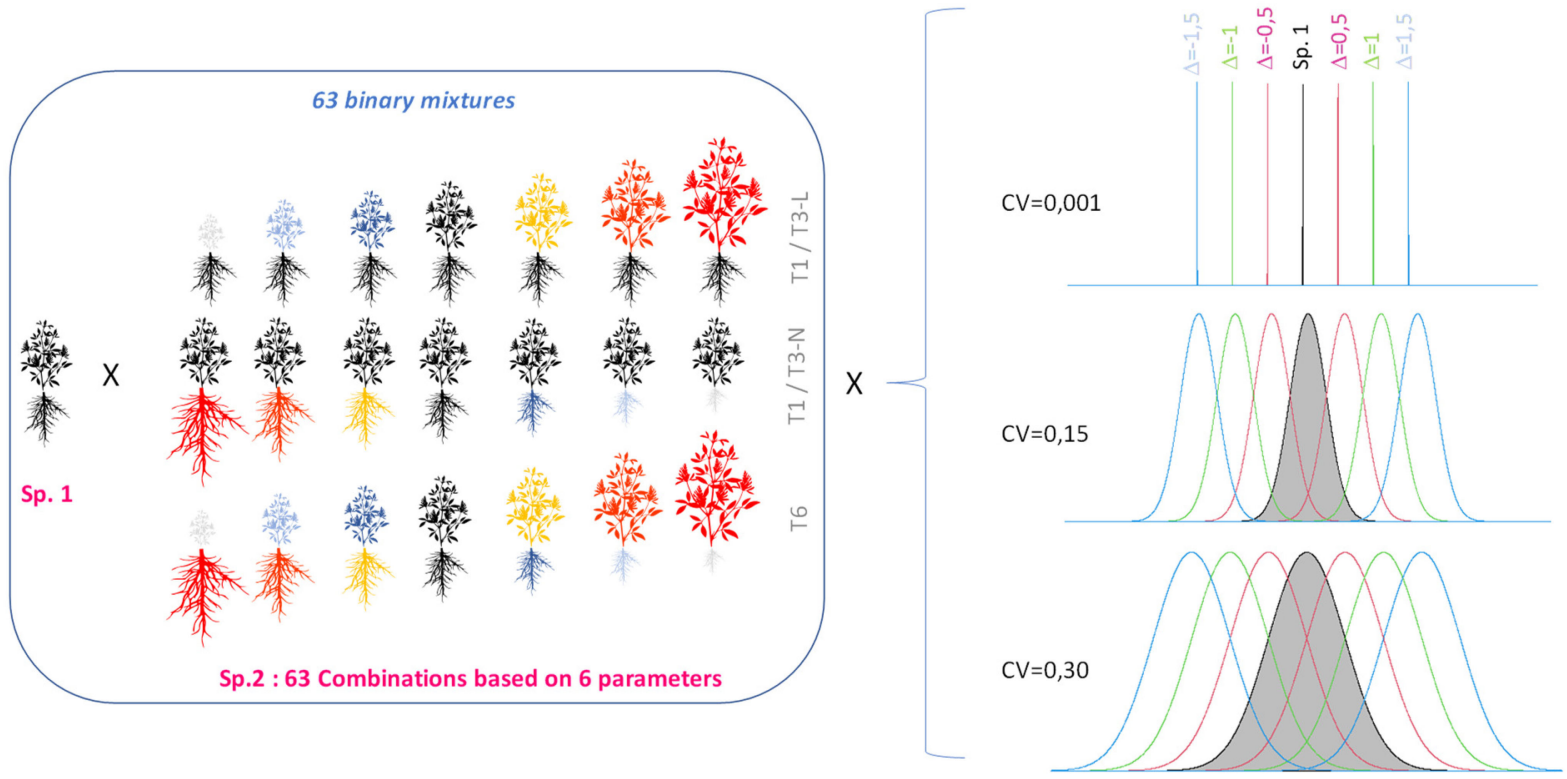
Design of the virtual experiment combining binary mixtures of a target species (Sp. 1, grey distribution) and a second species defined along a gradient of mean trait values (7 possible levels of Δ) and different sets of diverging parameters (1 to 6 parameters distinguishing Sp.2 either by traits for light acquisition only, or by traits for nitrogen acquisition, or finally a trade-off between access to both resources). A total of 63 plant communities were defined and three possible levels of within-species variation were considered (CV at 0.001, 0.15 or 0.30).

Each plant community was defined according to i) a level of mean trait divergence between species (Δ) and ii) a set of parameters (or ‘traits’) for which the two species differed (**Figure 1**). Seven possible levels of mean trait divergence (Δ) were defined (**Figure 1**; **Supplementary Figure B-a**). Not all possible combinations of the six parameters were included in the study and four possible trait combinations were more specifically investigated (T1, T3L, T3N and T6, differing by combinations of 1, 3 or 6 trait values); more details are shown in **Supplementary Tables B, C**. These scenarios included a series of situations at Δ equals zero (i.e. identical mean trait values between the mixed species) that corresponded to null models of interspecific competition.

In order to assess the impact of within-population parameter variation on interspecific competition, simulations with all 63 plant mixtures were run by testing the impact of the three possible CV levels previously defined: a very low (CV=0.001), moderate (CV=0.15) and high (CV=0.30) levels of intraspecific variation (**Figure 1**).

Finally, in order to assess the sensitivity of the conclusions to environmental variations, the entire set of plant communities was assessed under two contrasting pedoclimatic conditions. A first series of simulations was run under high soil N availability (N+, mineral N fertilisation set at 400 kg N ha^-1^, resulting in high light competition and limited N competition) and the second under low soil N availability (0N, no fertilisation, resulting in high N competition and more limited light competition). The initial conditions and other details of the simulations were as described in Louarn and Faverjon (2018) and corresponded dense forage plant stands (400 plants m^-2^), with a regular spacing and homogeneous initial soil conditions. They are further detailed in **Supplementary Table C**. Thus a total of approximately 7000 independent simulations were performed for this study. The simulations were repeated nine times for each combination of plant community, IV level and environment (N+/0N), in order to isolate the effects of stochastic processes in the model (e.g. affecting plant geometry) from those of the studied factors.

### 2.5 Model output analyses

**Figure 2:**
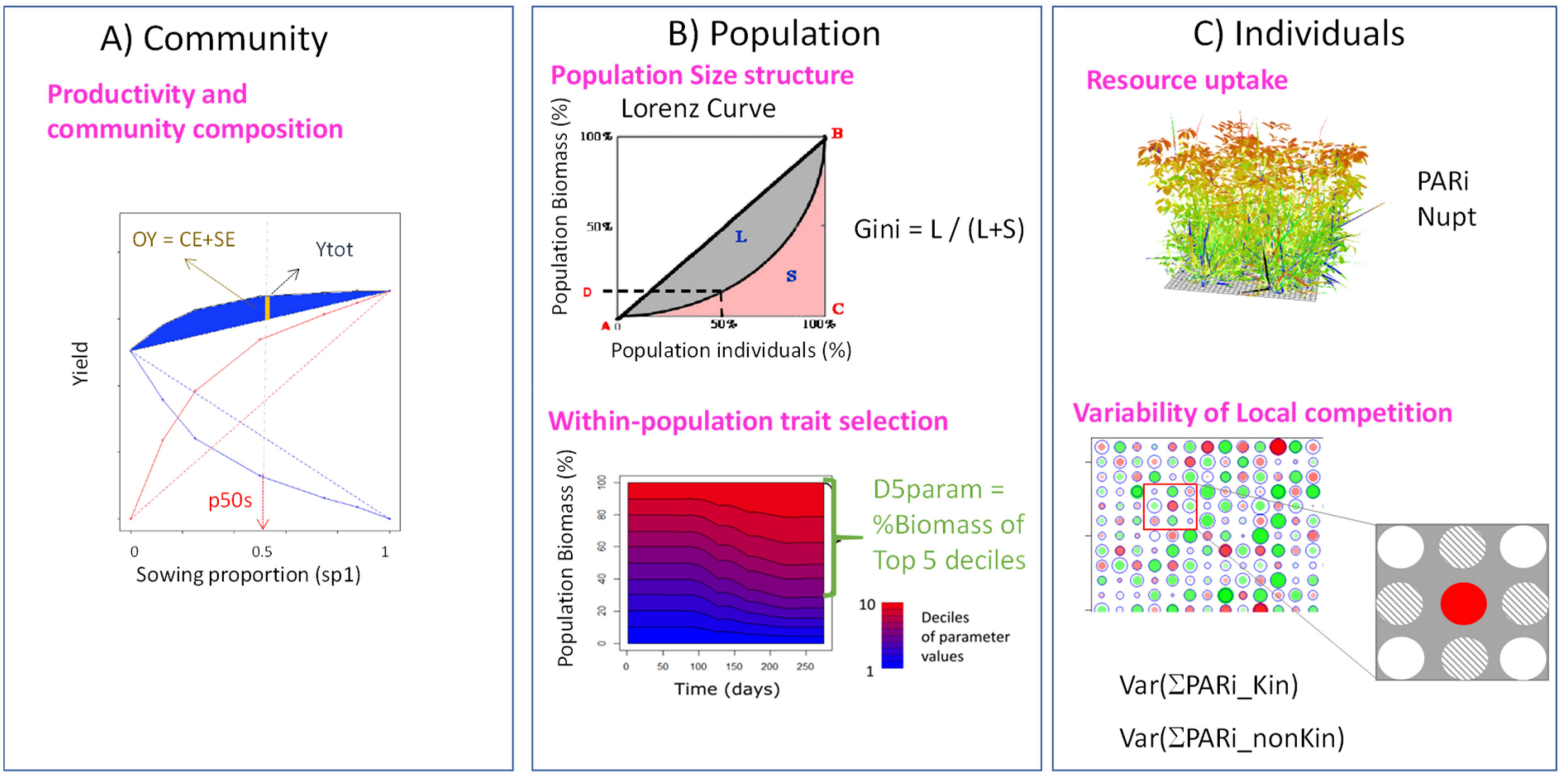
Illustration of the different indices used to characterise plant growth and competition for resources at different scales in binary mixtures, from whole canopy **(A)** to within-population criteria **(B)** and individual plant resource capture **(C)**. Ytot: Total annual production of the mixture; p50s: biomass proportion achieved over one year at 50/50 sowing; OY: overyielding; G: Gini coefficient; D5_param_: proportion of total population biomass represented by individuals in the top 5 deciles of values for a given parameter; PARi: cumulated amount of PAR intercepted per plant; Nupt: cumulated amount of mineral N absorbed per plant; Var(PARi_Kin_): variance of resource captured by local neighbours from the same species; Var(PARi_nonKin_): variance of resource captured by local neighbours from the other species.

The performance of binary mixtures was analysed using a range of indices that characterised plant growth and plant-plant interactions for resources at different scales (**Figure 2**). The objective was to take advantage of model outputs to analyse community performance and infer causal relationships between IV level on resource acquisition traits, state variables of individual plants accounting for their actual access to resources and the functioning of plant communities in terms of resource use and partitioning.

#### 2.5.1 Assessing mixture performance at the community level

First, at the whole community level, indices characterising agronomic performance were computed (**Figure 2A**). Total aboveground annual forage production cumulating all four harvests (Ytot = Yp_1,2_+Yp_2,1_; with Yp_1,2_ and Yp_2,1_ standing for partial yield of Sp1 growing with Sp2 and partial yield of Sp2 growing with Sp1, respectively) and overyielding (OY) of each possible binary mixture were determined to characterise community production:


(3) $$OY=Ytot–\left(Y_{pur1}+Y_{pur2}\right)/2$$

where Ypur1 and Ypur2 stand for the total annual yields of pure species which were simulated under the same environmental conditions.

OY has been shown to be separable into two additive components which characterise the contributions of resource use and changes in botanical composition to community performance (Loreau and Hector, 2001): the complementarity effect (CE) and the selection effect (SE). The CE component is derived from the change in the relative yield of species in the mixture (ΔRY) and the average production of all the species. In a binary mixture (n=2), it is calculated as:


(4) $$CE=n*\bar{RY}*\bar{Ypur}$$

The SE component is defined by the covariance between the production of pure species and the change in relative yield of species in the mixture:


(5) SE=n*Cov(RY,Ypur)

In addition, community stability was characterised for each mixture through the changes in species biomass proportions achieved over one year (p50s = Yp_2/_Ytot; Louarn et al., 2020), calculated between sowing and the last harvest. In such communities with a fixed number of species sown at equal proportions, p50s is theoretically equal to 0.5 for a mixture remaining stable over time.

Finally, in order to characterise the specific effect of IV on the balance between species in a given community, an index called the “stabilising effect” (S_IV_, unitless) was defined by comparing the p50s values of this particular community (i.e. same sets of plant parameters for Sp1 and Sp2) with and without IV. Taking the low IV (CV=0.001) as a reference, it was calculated as follows:


(6) $$\begin{array}{l} S_{IV}=\left(p50s_{LowIV}–0.5\right)–\left(p50s_{HighIV}–0.5\right)\;if\;p50s_{LowIV}\geq 0.5\\ S_{IV}=\left(0.5–p50s_{LowIV}\right)–\left(0.5–p50s_{HighIV}\right)\;if\;p50s_{LowIV}<0.5 \end{array}$$

Positive S_IV_ values indicate that IV contributed to remaining closer to the initial 50/50 sowing proportions, while negative values indicate on the contrary a greater shift in the proportion of species.

#### 2.5.2 Assessing IV effects at the population level

Secondly, indices were calculated to characterise within-species variability and the differential performance of individuals within each species (**Figure 2B**). The size structure and degree of asymmetry of plant populations were quantified using the Gini coefficient (G), which measures relative mean difference (i.e. the arithmetic average of differences between all pairs of surviving individuals; Weiner and Solbrig, 1984) and is frequently used as a proxy of competition intensity to qualify the degree of inequality of resource partitioning among individuals in a population (Weigelt and Jolliffe, 2003):


(7) $$G=(\sum_{i=1}^{n}\sum_{j=1}^{n}\left|x_{i}-x_{j}\right|)/\left(2n^{2}\bar{x}\right)$$

where *n* stands for the number of individuals in the population, i and j subscripts refer to distinct individuals from the same species, *x_i_* and *x_j_* refer to the plant biomasses of any given plant pair and $\bar{x}$ stands for the average plant biomass of the species. G values range from 0 (all individuals sharing resources equally) to 1 (all resources captured by a single individual). They were calculated separately for each species in a mixture (G_sp1_, G_sp2_).

In addition, a new population-level index was developed to assess the contribution of a given trait to within-species selection and link plant parameter values with the relative performance of individuals within a species (D5_param_). As for the Gini coefficient, this index was built on the basis of plant biomass distribution in the population, but it quantified the proportion of population biomass represented by the growth of individuals in the top 5 deciles of values of a given parameter. D5_param 1,2_ (standing for the *k* individuals of Sp1 growing with Sp2) was calculated as follows:


(8) $$D5_{param}1,2=(\sum_{i=1}^{k}xi)/Yp_{1,2}$$

where *xi* stands for individual plant biomass and Yp_1,2_ for the partial yield of Sp1 growing with Sp2. In the absence of any selection related to this parameter, the value for D5_param_ is expected to be 0.5, as the individuals in the first five deciles represent 50% of the initial population biomass in the seeds for all the simulated species and communities. D5_param_ values were compared at the end of the simulation period. This index was calculated for each of the six parameters used to define the plant communities (i.e. L_max_L, L_max_In, Phyllo_1_, Vmax_2_, EL_max_R and PPtresh_H_, for both Sp1 and Sp2). A rise in D5_param_ above 0.5 indicated that individuals with higher parameter values contributed relatively more to population productivity, and were thus positively selected within the species. Conversely, a fall in D5_param_ to below 0.5 indicated a counter-selection of individuals with the highest values for a given parameter.

#### 2.5.3 Assessing IV effects on the local access of individual plants to resources

Finally, the performance of individual plants was examined in terms of their effective capture of resources with respect to local neighbours, quantified from the cumulative amounts of light interception (*PARi*, standing for the photosynthetically active radiation intercepted) and soil mineral N uptake (*Nupt*) by each individual during the simulations. Resource capture was also analysed in terms of local competition, from the relative capture of resources by each plant with respect to its first-order neighbours. The variance of this local partitioning of resource between neighbours was used as an index of spatial heterogeneity within the canopy (**Figure 2C**). In this analysis, we distinguished between the effects of neighbours from the same (PARi_Kin_, intraspecific effects) or from the other species (PARi_nonKin_, interspecific effects) on the variance of local partitioning.

Statistical analyses were performed to analyse the effects of the main factor studied in the simulation design (namely, IV level and N fertility level, and mean trait divergence between species) on model outputs using R software (version 4.1.2; R Core Team, 2022). Significant differences between the means of a given output were tested by performing analyses of variance (*aov* procedure).

## 3 Results

### 3.1 Introducing the tested communities: impact of mean trait divergence between species on mixture yield and community stability at low IV

**Figure 3:**
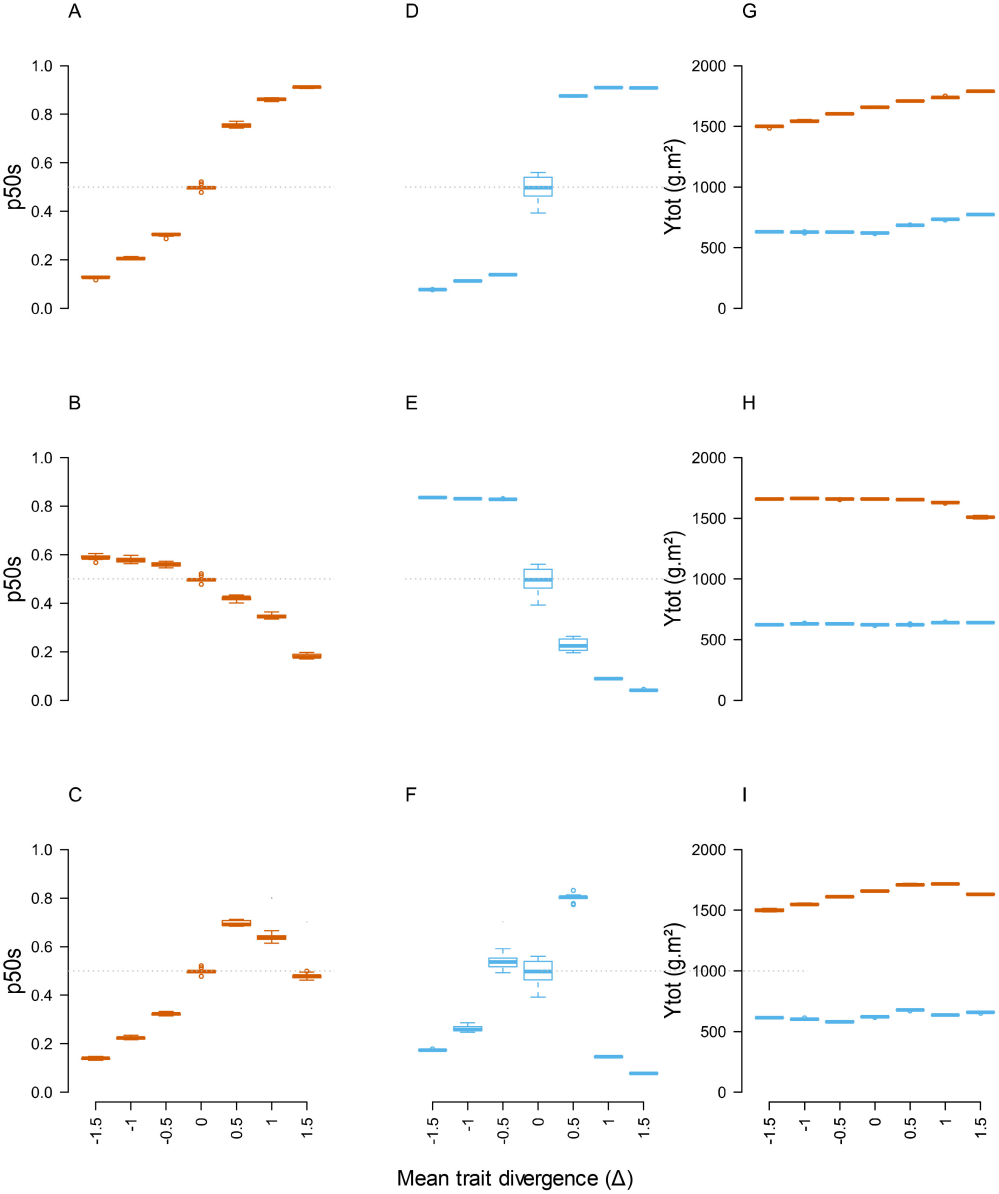
Responses of community stability (p50s) and total annual productivity (Ytot) to the gradient of mean trait divergence in T3L **(A, D, G)**, T3N **(B, E, H)** and T6 **(C, F, I)** communities with low within-species variation (CV=0.001). Blue and brown boxes indicate simulations under 0N and N+ environments, respectively. n=9 replicate simulations by community.

The impact of mean trait divergence (Δ) gradients was first examined in the absence of significant within-species parameter variation (CV=0.001). For the case of plant communities differing by parameters acting on a single plant function (T3L and T3N, **Figure 3**; T1, **Supplementary Figures B, C**), total annual productivity (Ytot) showed a relatively low response to mean trait divergence (e.g. from 1550 to 1680 g.m^-2^ on average at Δ =0 and Δ =+1.5 under high N for T1) and a strong response to soil N availability (from 550 to 1650 g.m^-2^ on average at low and high N). These values were close to the range covered by pure species in the same conditions (**Supplementary Table D**, from 960 to 1800 g.m^-2^ under high N, and from 430 to 770 g.m^-2^ under low N). By contrast, community stability (p50s) displayed a marked monotonous response to mean trait divergence, resulting in a shift from a stable situation (p50s approximately equal to 0.5 under all neutral scenarios and Δ=0) to a mixture markedly dominated by one or other species at Δ =+1.5 or Δ =-1.5 (e.g. p50s ranging from approximately 0.2 to 0.8 for species 2 (Sp2) under the most extreme scenarios). Quite remarkably, some parameters had more impact than others on the variations and ceiling values of p50s in T1 simulations (e.g. Phyllo_1_ and L_max_L > L_max_In for the light acquisition parameters; Vmax_2_ and EL_max_R > PPtresh_H_ for N uptake parameters). The relative impact of different parameters was also affected by N availability, with N uptake parameters having a markedly stronger impact on species balance under 0N than under N+ environment. A stronger impact of combined parameter changes was also noticeable in T3L and T3N compared to T1.

By contrast, scenarios differing by all 6 parameters (T6) and affecting different plant functions resulted in irregular responses of Ytot and p50s along the Δ gradient. Increasing mean trait divergence did not systematically lead to increasing differences in species proportions. In some cases, the outcome of multiple parameters acting on different dimensions of competitive ability could cause shifts of dominant species between moderate and high Δ values (e.g. under 0N, **Figure 3F**).

Overall, these patterns demonstrated that the 63 virtual plant communities we studied covered a wide range of situations in terms of plant-plant interactions and species balance. Despite significant differences in Ytot between the communities, the mixture yields were generally very close to the average yield of sole species and did not produce marked overyielding at low IV (OY< 50 g.m^-2^ in 120 out of 126 situations; **Supplementary Figure D**).

### 3.2 Impact of intraspecific variation in the case of null models of interspecific competition

**Figure 4:**
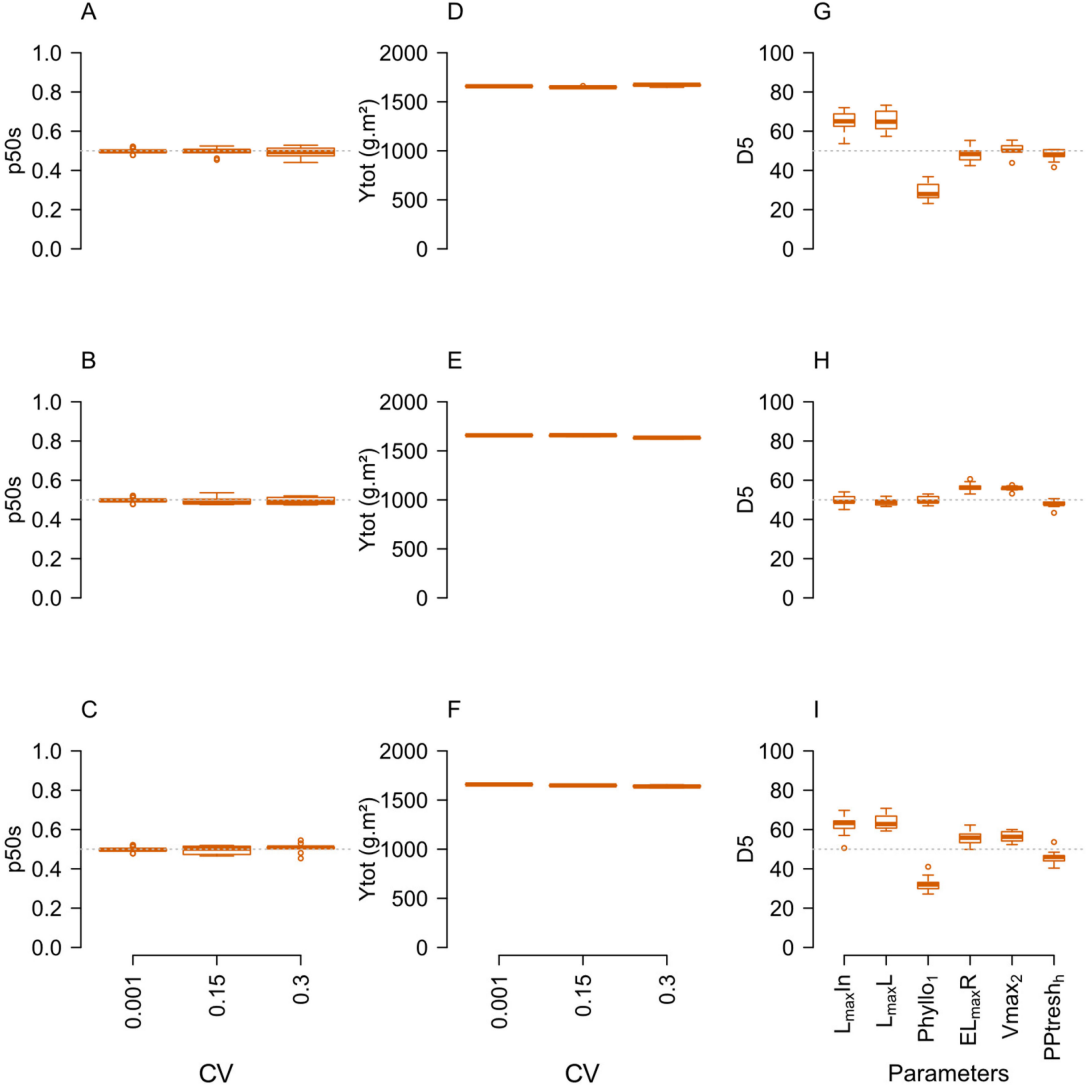
Responses of community stability (p50s, **A–C**) and total annual productivity (Ytot, **D–F**) to the IV level in situations corresponding to a null model of interspecific competition (Δ=0) in T3L (ADG), T3N (BEH) and T6 (CFI) communities under N+ and change in D5_param_ **(G–I)** for the different parameters at high IV (CV=0.30). n=9 replicate simulations by community.

The impact of within-species parameter variation (CV=0. 15 and CV=0.3) was first assessed in neutral situations of competition between species (i.e. Δ=0) for their impact at the population level. Quite remarkably, adding variance to one or more parameters had no impact on total yield and species balance (p50s equal to 0.5) in any of the neutral situations tested but strongly affected intra-specific competition (**Figure 4**; **Supplementary Figure E**). Increasing within-species variance indeed increased Gini coefficient (not shown) and the D5_param_ values (**Figures 4G–I**). In each community, parameters in which IV was increased showed deviations of D5_param_ from their initial 0.5 value (e.g. L_max_L, L_max_In and Phyllo_1_ in T3-L communities; Vmax_2_, EL_max_R and PPtresh_H_ in the T3-N communities; all parameters in T6 communities), while those set at a low IV remained close to 0.5 (e.g. L_max_L, L_max_In and Phyllo_1_ in T3-N communities; Vmax_2_, EL_max_R and PPtresh_H_ in T3-L communities). Even though the species proportions were not affected, the distribution of individual plant biomass was thus changed and individuals with a parameter value that favoured their relative competitive ability managed to produce a greater share of population biomass. Depending on the trait, this selection was associated with either increasing (L_max_L, L_max_In, Vmax_2_, EL_max_R) or decreasing (Phyllo_1_, PPtresh_H_) parameter values.

Interestingly, the magnitude of selection differed between parameters (e.g. the absolute shift of D5_param_ from 0.5 was higher for Phyllo_1_ > L_max_L > L_max_In for light acquisition parameters; Vmax_2_ and EL_max_R > PPtresh_H_ for N uptake parameters). The ranking of parameters offering a greater chance of within-species selection also changed depending on soil N availability (0N/N+; **Supplementary Figure B-e**).

### 3.3 Impact of intraspecific variation on mixture yield and community stability in contrasting communities

**Figure 5:**
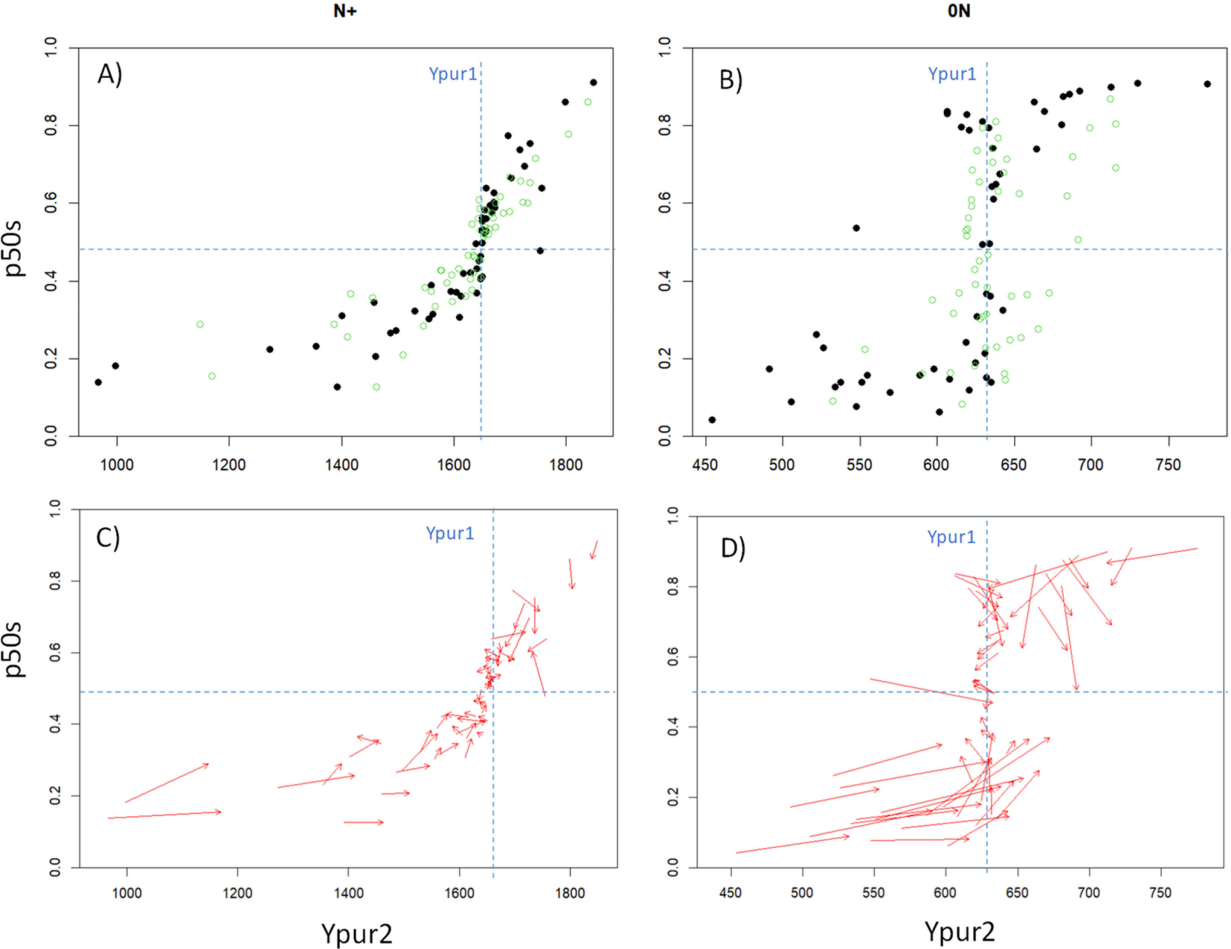
Relationship between the yield of pure species (Ypur2) and community stability (p50s) in mixture with Sp1 under high **(A–C)** and low **(B–D)** soil fertility for the whole set of virtual communities. Black and green dots represent communities at low (CV=0.001) and high (CV=0.3) IV, respectively. Red arrows **(C, D)** indicate the trajectories for each community when increasing IV. The intersections of blue dotted lines represent the null models of interspecific competition (Sp1 and Sp2 with identical mean parameter values) in each growing condition.

The impact of within-species parameter variation (CV=0. 15 and CV=0.3) was then assessed on all 63 plant communities. Overall, the relationship between the yield of pure species (Ypur2) and their proportion in mixture with Sp1 followed a non-linear increasing relationship under both N+ and 0N, with a central inflection point corresponding to the case of the null model of interspecific competition (**Figure 5**). Species with a pure yield relatively lower than that of Sp1 generally achieved p50s values below 0.5, and conversely for more productive species. The relationship was relatively monotonous under N+ and much noisier under 0N, denoting possible changes in the ranking of the 63 species between the different growing conditions. Interestingly, increasing IV caused significant changes in both the species yield and species proportions in the simulated mixtures (as indicated by arrows in **Figures 5C, D**). The general trend observed in both environments was to obtain communities closer to the case of null models, with improved community stability over time (i.e. p50s values closer to the initial 0.5 value under high IV) and reduced yield differences with Sp1.

**Figure 6:**
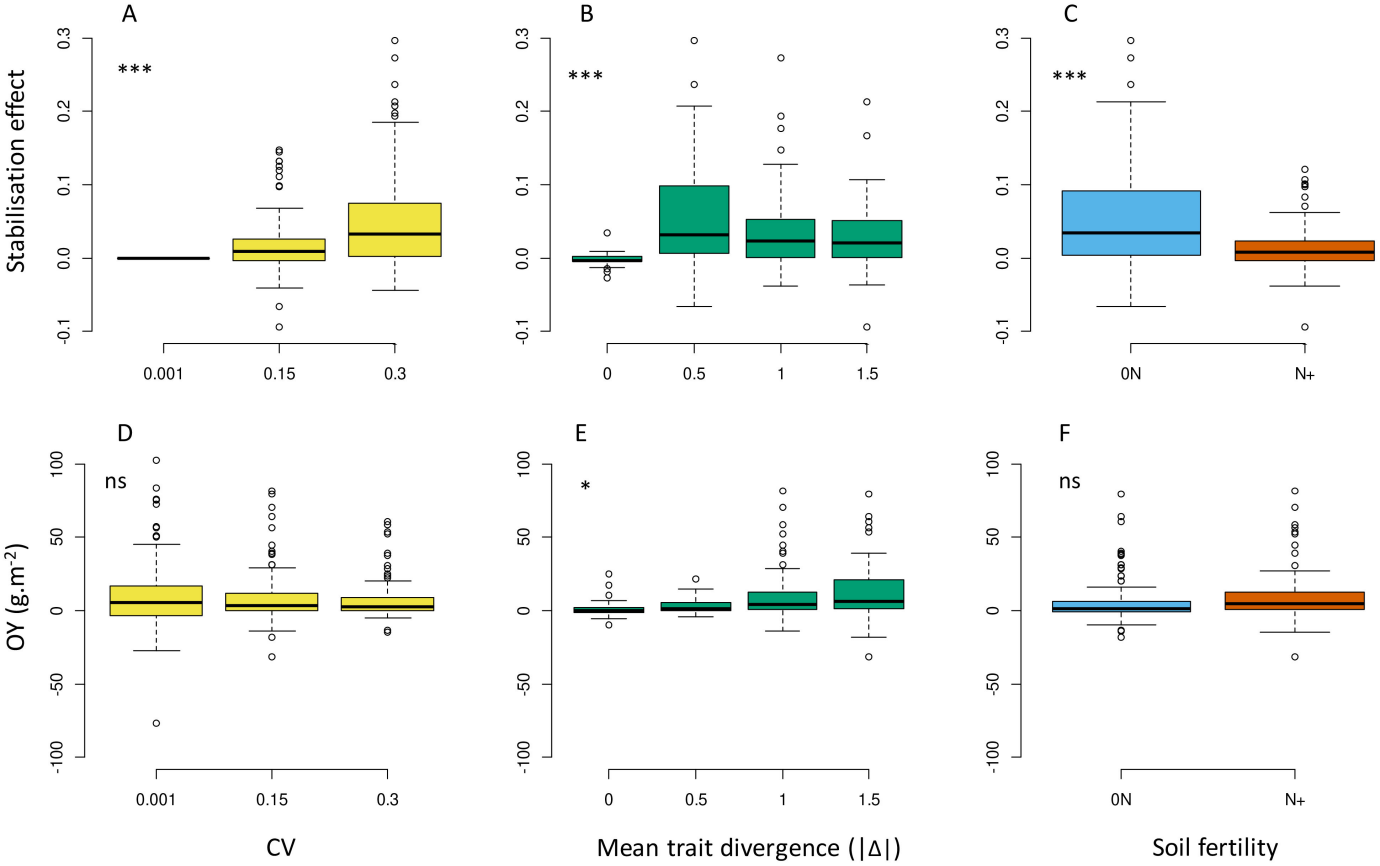
Effects of IV level **(A, D)**, absolute value of mean trait divergence **(B, E)** and soil fertility **(C, F)** on the stabilising effect of IV **(A–C)**, overyielding **(D–F)** predicted over the whole set of virtual communities. Stars indicate significant differences in means between treatments. ns: not significant. Overyielding (OY) and stabilising effect are calculated according to Equations 3, 6.

The stabilisation effect observed on p50s values (S_IV_) appeared greater at higher IV (i.e. modified up to 25% the final species proportion with CV=0.3; up to 13% with CV=0.15; **Figure 6A**) and under 0N (**Figure 6C**). S_IV_ was also highly variable among the tested communities at a given IV level and tended to be maximal at intermediate Δ values (i.e. close to the focal species Sp1, at -0.5 Δ or +0.5 Δ) and to decrease at high Δ values (e.g. -1.5 Δ or +1.5 Δ; **Figure 6B**). Regarding biomass production, increasing IV had no positive impact on mixture overyielding, with a general trend of OY being even closer to zero at high CV (**Figure 6D**; **Supplementary Figure B-b**). The detailed patterns of response of p50s and Ytot to Δ gradients are presented in the **Supplementary Material** (**Supplementary Figures F, B-c** for p50s; **Supplementary Figure B-b** for Ytot response). They were similar to that described at low IV in all combinations of parameters (T1, T3L, T3N and T6), but with improved community stability over time.

### 3.4 Impact of intraspecific variation on population size structure and within-species selection

**Figure 7:**
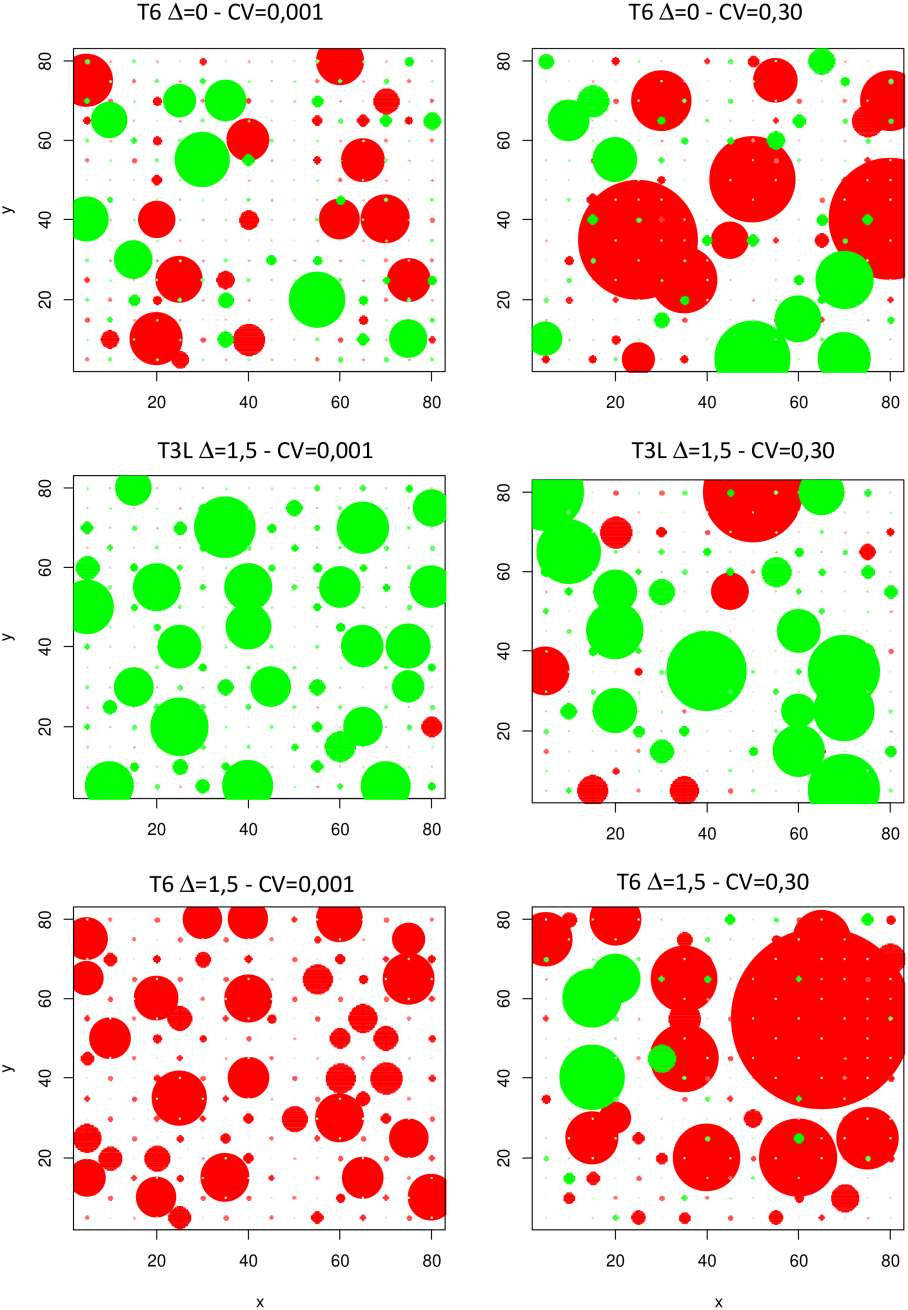
Spatial distribution of individual plant biomasses for Sp.1 (red circles) and Sp.2 (green circles) in three contrasting communities at low (CV=0.001) and high (CV=0.3) IV levels. Dot areas are proportional to plant biomass at the end of the simulations. Examples are for single run simulations under 0N.

**Figure 8:**
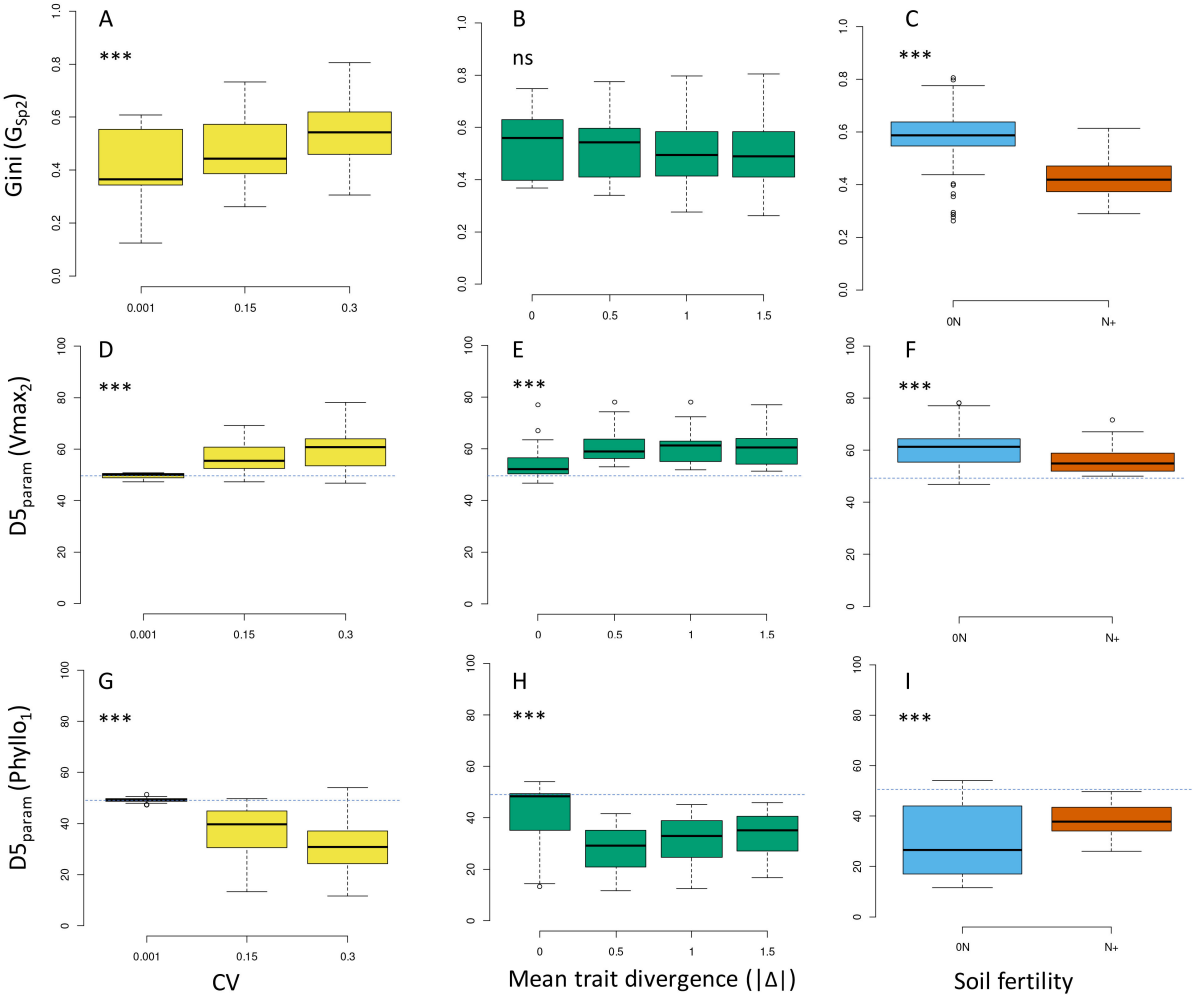
Effects of IV level **(A, D, G)**, absolute value of mean trait divergence **(B, E, H)** and soil fertility **(C, F, I)** on the Gini coefficient predicted for Sp2 (G_sp2_, **A–C**) and within-species selection index for Vmax_2_ (D5_param_Vmax2, **D–F**) and Phyllo_1_ parameters (D5_param_Phyllo1, **G–I**) in Sp2. Data are for the whole set of virtual communities for G_sp2_ and all the communities with diversity on Vmax_2_ and Phyllo_1_ parameters for D5_param_. *; **; *** indicate significant differences in means between treatments. ns: not significant. Dashed line: theoretical D5_param_ value in absence of within-species selection.

The consequences of increasing IV were also investigated in terms of within-species competition and demographic processes. The size distribution of individuals in the species studied were greatly affected with more parameter variations (**Figure 7**). As a result, inequality in size distribution and Gini coefficients increased significantly under moderate and high IV scenarios, irrespective of plant communities (**Figure 8A**; **Supplementary Figure B-d**). This indicated a higher share of total biomass produced by a smaller number of dominant individuals, and more unequal resource partitioning between individuals from the same species. This trend was observed in communities where Sp2 was dominant, subordinate or in a good balance with Sp1 (no effect of mean trait divergence, **Figure 8B**), and across all environments (0N, N+, **Figure 8C**), although more markedly under 0N.

The successful growth of individuals within a species did not occur at random and was related to their parameter values at moderate and high IV (**Supplementary Figure G**; **Supplementary Figure B-f**). Indeed, the parameter score (*P_score_*) of individual plants, which summarises the average parameter values of a given individual relative to its population, appeared to be correlated to plant biomass accumulation once a moderate (CV=0.15) or high IV (CV=0.3) was introduced into a population. Highly significant correlations between the biomass of individuals and their *P_score_* were found in these scenarios (r^2^ above 0.4 in 460 out of 504 cases, combining the different communities and considering both Sp1 and Sp2). Interestingly, this was not the case at low IV (r^2^ close to 0 at CV=0.001), in a situation where all plants of a species had lower range of parameter values, and where initial random conditions in the model explained most of the differences in biomass production between plants.

Within-species selection signatures were also apparent from shifts of D5_param_ values at moderate and high IV (**Figures 8D–G**; **Supplementary Figure B-e**). Indeed, individuals with higher (respectively lower) parameter values contributed more to total population biomass when providing a competitive advantage. The magnitude of selection (as indicated by the absolute shift of D5_param_ from 0.5) differed depending on the trait, environment (more important under 0N, **Figures 8F–I**) and community considered (**Figures 8E–H**). The most important traits could differ depending on the growing conditions. For instance, greater internode length (L_max_In, **Supplementary Figure H-ACEG**) was systematically selected in focal species Sp1 in N+ environments but not in 0N environments. Conversely, a higher root elongation rate (EL_max_R, **Supplementary Figure H-BDFH**) was systematically selected in 0N environments, but only in some communities under N+ conditions.

### 3.5 Relationship with plant interactions for light and N

**Figure 9:**
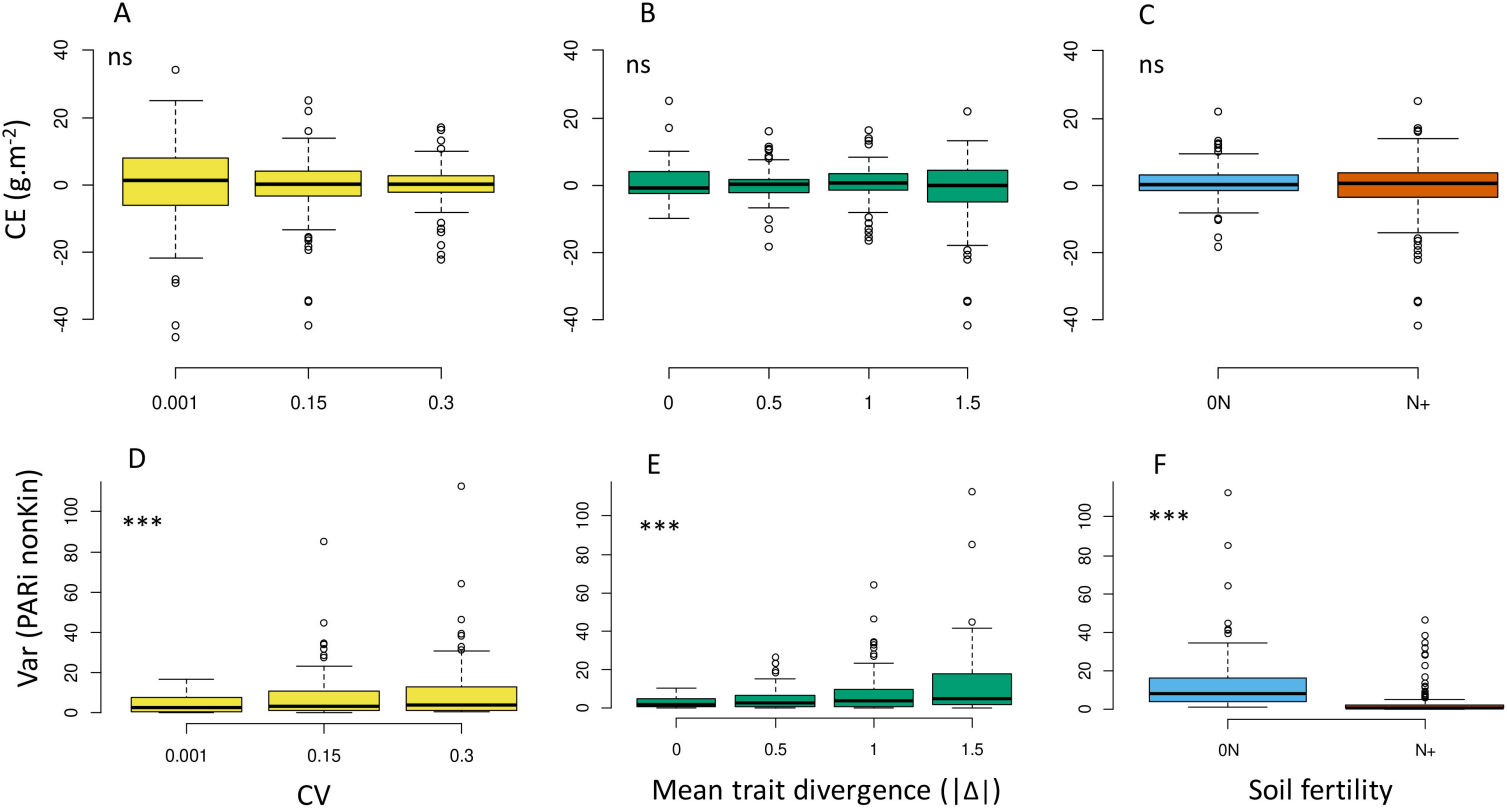
Effects of IV level **(A, D)**, absolute value of mean trait divergence **(B, E)** and soil fertility **(C, F)** on Loreau & Hector’s complementarity effect **(A–C)** and the spatial heterogeneity of resource captured by first-order neighbours from other species (**D–F**, variance of PARi_nonKin_) predicted over the whole set of virtual communities. *; **; *** indicate significant differences in means between treatments. ns, not significant. Complementarity effect (CE) is calculated according to Equation 5.

At the community level, increasing IV did not result in higher complementarity effect (CE, **Figure 9A**; **Supplementary Figure B-g**) and thus did not cause more resources to be captured or an improvement in the use of these resources by the tested communities. In line with OY, its two components CE and SE (not shown) tended to be closer to zero at high CV, with no significant impact of mean trait divergence and soil fertility level (**Figures 9B, C**).

At a local plant-plant interaction level, the temporal dynamics of resource partitioning between neighbour plants, and their relationship with differential growth between species, were also examined. As expected from model formalisms, individual plant biomass accumulation was driven directly by light interception and N uptake, irrespective of the species and virtual communities (**Supplementary Figure I**; **Supplementary Figure B-f**). Cumulative *PARi* was linearly related and strictly proportional to plant biomass under N+ and 0N, although with a different slope and a slightly lower correlation coefficient under 0N. The level of resource capture almost entirely explained inter-individual variations in biomass accumulation both within and between species, irrespective of IV levels (r^2^>0.98 in 756 out of 756 possible combinations). *Nupt* was also strongly related to plant biomass, but usually with a lower correlation (r^2^>0.94 in 756 out of 756 possible combinations; **Supplementary Figure G-c**; **Supplementary Figure B-f**).

The partitioning of resource between neighbour plants was modified by IV treatments and affected the strength of local competitive interactions (**Figures 9D–F**). Increasing IV resulted in higher within-canopy variance of local light competition, from both conspecifics and neighbours from the other species (e.g. higher median and broader ranges of within-canopy PARi_nonKin_ variances; **Figure 9D**). This index, which reflected the spatial heterogeneity of competitive interactions, was generally of a much higher magnitude under 0N and for interspecific competition in communities at high Δ values.

## 4 Discussion

### 4.1 Relative effects of mean trait divergence and IV on the total production and community stability of simulated forage mixtures

This simulation study highlighted the value of mechanistic models that explicitly simulate plant-plant interactions i) to analyse the effects of intraspecific genetic variation on resource partitioning and use at different organisational levels (i.e. individual, population, community), ii) to assess the potential relationships explaining these patterns and iii) to identify the plant traits and levels of genetic variation with the greatest impact on the desired properties of forage mixtures. The simulation results were in line with previous observations of the positive effects of IV on the stability of community composition, but not necessarily on total community productivity, as reported in grassland diversity experiments (Williams et al., 2003; Fridley and Grime, 2010; Meilhac et al., 2019). Over the range of virtual communities tested, increasing IV even at a moderate level resulted in more stable species proportion over time. However, no favourable impact on total forage production was noted, with even a general trend of being closer to the average yield of sole species. This lack of overyielding might not be a general feature of genetically diversified systems, but circumstantial to the choice of traits and species considered to design our mixtures. For instance, the inclusion of legume species enables strong niche differentiation for N in grasslands, and is usually associated with significant overyielding (Nyfeler et al., 2009; Lüscher et al., 2014; Louarn et al., 2020). Here, only traits involved in light and mineral nitrogen capture were considered, which offered limited possibilities for resource substitution through their combinations. Complementarities could mostly occur from differences in the spatial and temporal use of light and soil N, as in grass species mixtures (Pontes et al., 2012; Cougnon et al., 2014).

The simulation design also allowed us to assess the strength of IV effects with respect to species mixtures defined along a mean trait divergence gradient (Δ). The hypothesis of a stronger IV effect on communities with overlapping species trait values was supported by our results (**Figure 6B**). Indeed, the stabilising effect of IV was not constant over the different Δ levels and had a maximal span in communities with moderately divergent species (+/-0.5 Δ), tending to decrease thereafter with the most divergent mixtures. The significance of IV to competition in forage mixtures may thus preferably concern species with close ecological niches and relatively similar morphologies and growth pattern. The IV effect appeared positive for community stability in most situations tested, but it was generally much weaker than effects induced by mean trait divergence. For instance, the strongest IV effect in our simulation (at CV=0.3, S_IV_ reached 0.05 in average) compensated for only half of the lowest Δ effects on changes in species proportion (at |Δ|=0.5 and low IV, p50s were above 0.6 or below 0.4, equivalent to a minimum 0.10 deviation from the initial proportions). This lower magnitude was consistent with previous experiments (Crutsinger et al., 2009) but the actual quantitative difference with respect to mean traits effects may depend on the species, traits and time frame considered (Albert et al., 2010; Meilhac et al., 2019; Montazeaud et al., 2020).

Overall, the positive effects of IV observed in this study confirmed the potential of this management option in forage mixtures, and the feasibility of a model-assisted approach to identifying relevant traits and IV levels. However, the actual impact in real mixtures will depend on the forage species targeted (each with its own potential pool of genetic diversity), the traits considered (including functions not considered in this study: disease resistance, phenology, abiotic stress, etc.) and environmental conditions.

### 4.2 Major traits involved and sensitivity to environmental stressors

A second hypothesis tested during this study concerned the importance of environmental stressors to the expression of IV effects. The same 63 communities grown under high and low soil N fertility had contrasting growth and differential responses to increasing IV. Total productivity was obviously improved under N+, but did not affect the OY levels. On the other hand, communities under N stress were more unbalanced in terms of species proportion and responded better to increasing IV, with more pronounced stabilising effects (**Figure 6C**). These results were consistent with field observations highlighting the greater benefits of IV under N stress (Jokinen, 1991) and other stressors (e.g. Reiss and Drinkwater, 2018 for a review on cultivar mixtures; Clark, 2010 for the highest impact of IV in forest communities under drought).

All the traits chosen to build our virtual communities were related to light and N acquisition, and selected because of the sensitivity of the model outputs to their values. Accordingly, divergences of mean parameter values between species all produced effects on community composition, but not all of the same intensity. Interestingly, they all also proved sensitive to increasing IV beyond moderate values, with the selection of individuals with the fittest parameter values within populations (as shown by the predicted shifts in D5_param_). As expected from the theory (Damgaard, 2004), the genotypes favoured under each growing condition presented different phenotypic profiles, with directional selection for traits involved in higher N acquisition under 0N, and conversely for traits improving light acquisition under N+. Thus, traits controlling different functions could be affected differentially depending on the most limiting resource (Hetzer et al., 2021), with individual plant growth improving through its capture. Some traits with diversity offered few advantages in a particular environment and were not selected in that environment (e.g. L_max_In under 0N), whereas others were important for the capture of both resources and were selected in all environments (e.g. those related to growth kinetics: Phllo_1_). Such short-term selection of genotypic composition has previously been reported within grassland populations, and may concern adaptation to local abiotic conditions (e.g. winter survival, Collins et al., 2001, 2012) and co-selection with neighbouring characteristics within plant communities (Aarssen and Turkington, 1985; Lüscher et al., 1992; Verwimp et al., 2018; Ergon et al., 2019). Compared to such empirical results with molecular markers, an added value of modelling studies is that it provides an opportunity to directly assess which traits may potentially be involved and could have the strongest impact on adaptation (Verwimp et al., 2018). Clearly, further work is now needed to address the numerous questions raised by multi-trait differences within actual forage mixtures and to investigate in further details trade-offs in plant functioning (e.g. growth versus defence, Albrecht and Argueso, 2017) or genetic constraints (e.g. involving genetic correlations between traits, Chen and Lübberstedt, 2010; Picheny et al., 2017).

### 4.3 Possible mechanisms underlying the “stabilising” effect of IV: complementarity versus spatial heterogeneity in local interactions?

The most straightforward effects of increased IV in this study concerned its impact on the community stability. This was associated with shifts in the genotypic composition of species that involved different sets of traits depending on the communities, and depended on selective pressures in its environment (light versus N limitation). Several mechanisms have been suggested that might explain this type of pattern (Bolnick et al., 2011; Ehlers et al., 2016; Snyder et al., 2020), but they are often difficult to quantify and isolate in empirical observations and experiments. Because a process-based model offers access to resource capture by plants, simulation controls and many intermediate variables, we were able to investigate the potential contribution of different mechanisms.

A common assumption made about the positive effects of IV concerns a potential increase in the niche breadth of species that allows greater complementarity between species (Litrico and Violle, 2015; Prieto et al., 2015). As discussed above, competitive interactions tended to dominate plant-plant interactions in most tested communities and the limited OY and Loreau-Hector’s complementarity index (CE, Loreau and Hector, 2001) suggested quite a limited role for improved species complementarity in the patterns we observed.

Alternatively, a clear causal relationship was found between individual access to resources (PARi and Nupt), differential growth with neighbours and genetic plant parameter values (P_score_). Our study provided evidence that increasing IV could result in a relative increase in competition intensity with conspecifics while reducing competition with the other species, as originally hypothesised by Clark (2010). Concomitant changes in the size hierarchy within each species (increased Gini coefficients) and towards more balanced binary mixtures supported this view in most non-neutral communities. An impact of IV on spatial heterogeneity of the canopy formed by the dominant species, offering more suitable micro-environments for the development of subordinate species (Ehlers et al., 2016), was consistently observed across the communities tested. Such a ‘competitor release’ effect at the community level could contribute to creating gaps in the control of resource capture by the dominant species and open resource patches for the subordinate species (Hughes et al., 2008). The greater spatial heterogeneity of interspecific competition thus resulted in a higher probability of recruiting dominant individuals within the subordinate species, especially when the differential of competitive ability between species was low (i.e. intermediate Δ).

Overall, relatively ubiquitous impacts of IV on temporal community stability were noted and were expressed more intensely in forage plant communities with moderate mean trait divergence and under stressful environmental conditions. The results obtained with a spatially-explicit IBM simulating resource partitioning above and belowground allowed us to infer the role of different traits and IV levels in different environmental conditions. The mechanisms identified for conditions typical of grasslands without legumes were linked to changes in resource partitioning rather than impacts on the total amount of available resources or increased complementarity between species. These results highlight the potential usefulness of IV to the design of improved forage production systems. They also illustrate how models might be helpful to specify the advantageous trait combinations on which breeders and farmers should focus in order to adapt genetic material (Bourke et al., 2021) and assembly rules (Evers et al., 2019; Stomph et al., 2020) to perform well with mixtures in low-input environments.

## Data Availability

The datasets presented in this study can be found in online repositories. The names of the repository/repositories and accession number(s) can be found in the article/**Supplementary Material**.

## References

[B1] Aarssen L. W. Turkington R. (1985). Biotic specialization between neighbouring genotypes in Lolium perenne and Trifolium repens from a permanent pasture. J. Ecol. 73 (2), 605–614. doi: 10.2307/2260497

[B2] Albert C. H. Thuiller W. Yoccoz N. G. Douzet R. Aubert S. Lavorel S. (2010). A multi-trait approach reveals the structure and the relative importance of intra-vs. interspecific variability in plant traits. Funct. Ecol. 24, 1192–1201. doi: 10.1111/j.1365-2435.2010.01727.x

[B3] Albrecht T. Argueso C. T. (2017). Should I fight or should I grow now? The role of cytokinins in plant growth and immunity and in the growth–defence trade-off. Ann. Bot. 119, 725–735. doi: 10.1093/aob/mcw211 27864225 PMC5379597

[B4] Annicchiarico P. Collins R. P. De Ron A. M. Firmat C. Litrico I. Hauggaard-Nielsen H. (2019). Do we need specific breeding for legume-based mixtures? Adv. Agron. 157, 141–215. doi: 10.1016/bs.agron.2019.04.001

[B5] Baldissera T. C. Frak E. Carvalho P. C. D. F. Louarn G. (2014). Plant development controls leaf area expansion in alfalfa plants competing for light. Ann. Bot. 113 (1), 145–157. 24201140 10.1093/aob/mct251 PMC3864722

[B6] Barillot R. Escobar-Gutiérrez A. J. Fournier C. Huynh P. Combes D. (2014). Assessing the effects of architectural variations on light partitioning within virtual wheat–pea mixtures. Ann. Bot. 114, 725–737. doi: 10.1093/aob/mcu099 24907314 PMC4217680

[B7] Barot S. Allard V. Cantarel A. Enjalbert J. Gauffreteau A. Goldringer I.. (2017). Designing mixtures of varieties for multifunctional agriculture with the help of ecology. A review Agron. Sustain. Dev. 37, 1–20. doi: 10.1007/s13593-017-0418-x

[B8] Becker C. Berthomé R. Delavault P. Flutre T. Fréville H. Gibot-Leclerc S.. (2022). The ecologically relevant genetics of plant-plant interactions. Trends Plant Sci. 28 (1), 31–42. doi: 10.1016/j.tplants.2022.08.014 36114125

[B9] Bedoussac L. Journet E. P. Hauggaard-Nielsen H. Naudin C. Corre-Hellou G. Jensen E. S.. (2015). Ecological principles underlying the increase of productivity achieved by cereal-grain legume intercrops in organic farming. A review. Agron. Sustain Dev. 35, 911–935. doi: 10.1007/s13593-014-0277-7

[B10] Blanc E. Barbillon P. Fournier C. Lecarpentier C. Pradal C. Enjalbert J. (2021). Functional–structural plant modeling highlights how diversity in leaf dimensions and tillering capability could promote the efficiency of wheat cultivar mixtures. Front. Plant Sci. 12. doi: 10.3389/fpls.2021.734056 PMC8511389 34659301

[B11] Bolnick D. I. Amarasekare P. Araújo M. S. Bürger R. Levine J. M. Novak M.. (2011). Why intraspecific trait variation matters in community ecology. Trends Ecol. Evol. 26, 183–192. doi: 10.1016/j.tree.2011.01.009 21367482 PMC3088364

[B12] Booth R. E. Grime J. P. (2003). Effects of genetic impoverishment on plant community diversity. J. Ecol. 91, 721–730. doi: 10.1046/j.1365-2745.2003.00804.x

[B13] Borg J. Kiær L. P. Lecarpentier C. Goldringer I. Gauffreteau A. Saint-Jean S.. (2018). Unfolding the potential of wheat cultivar mixtures: A meta-analysis perspective and identification of knowledge gaps. Field Crops Res. 221, 298–313. doi: 10.1016/j.fcr.2017.09.006

[B14] Bourke P. M. Evers J. B. Bijma P. Van Apeldoorn D. Smulders M. J. Kuyper T.. (2021). Breeding beyond monoculture: putting the’intercrop’into crops. Front. Plant Sci. 12, 734167. doi: 10.3389/fpls.2021.734167 34868116 PMC8636715

[B15] Brooker R. W. Bennett A. E. Cong W. F. Daniell T. J. George T. S. Hallett P. D.. (2015). Improving intercropping: a synthesis of research in agronomy, plant physiology and ecology. New Phytol. 206, 107–117. doi: 10.1111/nph.13132 25866856

[B16] Brummer E. C. Moore K. J. Bjork N. C. (2002). Agronomic consequences of dormant–nondormant alfalfa mixtures. Agron. J. 94, 782–785. doi: 10.2134/agronj2002.0782

[B17] Chen Y. Lübberstedt T. (2010). Molecular basis of trait correlations. Trends Plant Sci. 15, 454–461. doi: 10.1016/j.tplants.2010.05.004 20542719

[B18] Clark J. S. (2010). Individuals and the variation needed for high species diversity in forest trees. Science 327, 1129–1132. doi: 10.1126/science.1183506 20185724

[B19] Clark J. S. Bell D. M. Hersh M. H. Kwit M. C. Moran E. Salk C.. (2011). Individual-scale variation, species-scale differences: inference needed to understand diversity. Ecol. Lett. 14, 1273–1287. doi: 10.1111/j.1461-0248.2011.01685.x 21978194

[B20] Collins R. P. Helgadóttir Á. Fothergill M. Rhodes I. (2001). Variation amongst survivor populations of two white clover cultivars collected from sites across Europe: morphological and reproductive traits. Ann. Bot. 88, 761–770. doi: 10.1006/anbo.2001.1462 PMC4233816 12096740

[B21] Collins R. P. Helgadóttir Á. Frankow-Lindberg B. E. Skøt L. Jones C. Skøt K. P. (2012). Temporal changes in population genetic diversity and structure in red and white clover grown in three contrasting environments in northern Europe. Ann. Bot. 110, 1341–1350. doi: 10.1093/aob/mcs058 22437665 PMC3478043

[B22] Cougnon M. Baert J. Van Waes C. Reheul D. (2014). Performance and quality of tall fescue (F estuca arundinacea S chreb.) and perennial ryegrass (L olium perenne L.) and mixtures of both species grown with or without white clover (T rifolium repens L.) under cutting management. Grass Forage Sci. 69, 666–677. doi: 10.1111/gfs.12102

[B23] Crutsinger G. M. Collins M. D. Fordyce J. A. Gompert Z. Nice C. C. Sanders N. J. (2006). Plant genotypic diversity predicts community structure and governs an ecosystem process. science 313, 966–968. doi: 10.1126/science.1128326 16917062

[B24] Crutsinger G. M. Sanders N. J. Classen A. T. (2009). Comparing intra-and inter-specific effects on litter decomposition in an old-field ecosystem. Basic Appl. Ecol. 10, 535–543. doi: 10.1016/j.baae.2008.10.011

[B25] Damgaard C. (2004). Evolutionary Ecology of Plant-Plant Interactions – An Empirical Modelling Approach (Aarhus, DK: Aarhus University Press).

[B26] Ehlers B. K. Damgaard C. F. Laroche F. (2016). Intraspecific genetic variation and species coexistence in plant communities. Biol. Lett. 12, 20150853. doi: 10.1098/rsbl.2015.0853 26790707 PMC4785922

[B27] Ergon Å. Skøt L. Sæther V. E. Rognli O. A. (2019). Allele frequency changes provide evidence for selection and identification of candidate loci for survival in red clover (Trifolium pratense L.). Front. Plant Sci. 10, 718. doi: 10.3389/fpls.2019.00718 31244867 PMC6580991

[B28] Evans D. R. Williams T. A. Jones S. Evans S. A. (1995). The effect of blending white clover varieties and their contribution to a mixed grass/clover sward under continuous sheep stocking. Grass Forage Sci. 50, 10–15. doi: 10.1111/j.1365-2494.1995.tb02288.x

[B29] Evers J. B. van der Werf W. Stomph T. J. Bastiaans L. Anten N. P. (2019). Understanding and optimizing species mixtures using functional–structural plant modelling. J. Exp. Bot. 70, 2381–2388. doi: 10.1093/jxb/ery288 30165416

[B30] Faverjon L. Escobar-Gutiérrez A. Litrico I. Julier B. Louarn G. (2019). A generic individual-based model can predict yield, nitrogen content, and species abundance in experimental grassland communities. J. Exp. Bot. 70, 2491–2504. doi: 10.1093/jxb/ery323 30219923

[B31] Faverjon L. Escobar-Gutiérrez A. J. Litrico I. Louarn G. (2017). A conserved potential development framework applies to shoots of legume species with contrasting morphogenetic strategies. Front. Plant Sci. 8, 405. doi: 10.3389/fpls.2017.00405 28396676 PMC5366346

[B32] Fisher R. A. (1918). The correlation between relatives on the supposition of mendelian inheritance. Trans. R. Soc Edinb. 53, 399–3433.

[B33] Fridley J. D. Grime J. P. (2010). Community and ecosystem effects of intraspecific genetic diversity in grassland microcosms of varying species diversity. Ecology 91, 2272–2283. doi: 10.1890/09-1240.1 20836449

[B34] Gaba S. Lescourret F. Boudsocq S. Enjalbert J. Hinsinger P. Journet E. P.. (2015). Multiple cropping systems as drivers for providing multiple ecosystem services: from concepts to design. Agron. Sustain. Dev. 35, 607–623. doi: 10.1007/s13593-014-0272-z

[B35] Gallais A. (1992). Pourquoi faire des variétés synthétiques? Agronomie 12, 601–609. doi: 10.1051/agro:19920803

[B36] Gaudio N. Louarn G. Barillot R. Meunier C. Vezy R. Launay M. (2022). Exploring complementarities between modelling approaches that enable upscaling from plant community functioning to ecosystem services as a way to support agroecological transition. silico Plants 4, diab037. doi: 10.1093/insilicoplants/diab037

[B37] Gautier H. Měch R. Prusinkiewicz P. Varlet-Grancher C. (2000). 3D architectural modelling of aerial photomorphogenesis in white clover (Trifolium repens L.) using L-systems. Ann. Bot. 85, 359–370. doi: 10.1006/anbo.1999.1069

[B38] Harris W. (2001). Formulation of pasture seed mixtures with reference to competition and succession in pastures," in Competition and succession in pastures. Eds. Tow P. G. Lazenby A., (New York: CABI Publishing) pp. 149–174.

[B39] Hetzer J. Huth A. Taubert F. (2021). The importance of plant trait variability in grasslands: a modelling study. Ecol. Model. 453, 109606. doi: 10.1016/j.ecolmodel.2021.109606

[B40] Hughes A. R. Inouye B. D. Johnson M. T. Underwood N. Vellend M. (2008). Ecological consequences of genetic diversity. Ecol. Lett. 11, 609–623. doi: 10.1111/j.1461-0248.2008.01179.x 18400018

[B41] Jokinen K. (1991). Influence of different barley varieties on competition and yield performance in barley-oats mixtures at two levels of nitrogen fertilization. AFSci 63, 341–351. doi: 10.23986/afsci.72407

[B42] Julier B. Huyghe C. Ecalle C. (2000). Within-and among-cultivar genetic variation in alfalfa: Forage quality, morphology, and yield. Crop Sci. 40, 365–369. doi: 10.2135/cropsci2000.402365x

[B43] Justes E. Bedoussac L. Dordas C. Frak E. Louarn G. Boudsocq S.. (2021). The 4C approach as a way to understand species interactions determining intercropping productivity. Front. Agric. Sci. Eng. 8 (3), 3. doi: 10.15302/J-FASE-2021414

[B44] Kammoun B. Journet E. P. Justes E. Bedoussac L. (2021). Cultivar grain yield in durum wheat-grain legumes intercrops could be estimated from sole crop yields and interspecific interaction index. Front. Plant Sci. 12, 733705. doi: 10.3389/fpls.2021.733705 34721461 PMC8551613

[B45] Krzanowski W. J. (2000). Principles of multivariate analysis: a user’sperspective (New York: Oxford University Press).

[B46] Li L. Tilman D. Lambers H. Zhang F.-S. (2014). Plant diversity and overyielding: insights from belowground facilitation of intercropping in agriculture. New Phytol. 203, 63–69. doi: 10.1111/nph.12778 25013876

[B47] Li S. van der Werf W. Zhu J. Guo Y. Li B. Ma Y.. (2021). Estimating the contribution of plant traits to light partitioning in simultaneous maize/soybean intercropping. J. Exp. Bot. 72, 3630–3646. doi: 10.1093/jxb/erab077 33608704

[B48] Litrico I. Violle C. (2015). Diversity in plant breeding: a new conceptual framework. Trends Plant Sci. 20, 604–613. doi: 10.1016/j.tplants.2015.07.007 26440430

[B49] Loreau M. Hector A. (2001). Partitioning selection and complementarity in biodiversity experiments. Nature 412, 72–76. doi: 10.1038/35083573 11452308

[B50] Louarn G. Barillot R. Combes D. Escobar-Gutiérrez A. (2020). Towards intercrop ideotypes: non-random trait assembly can promote overyielding and stability of species proportion in simulated legume-based mixtures. Ann. Bot. 126, 671–685. doi: 10.1093/aob/mcaa014 32004372 PMC7489071

[B51] Louarn G. Faverjon L. (2018). A generic individual-based model to simulate morphogenesis, C–N acquisition and population dynamics in contrasting forage legumes. Ann. Bot. 121, 875–896. doi: 10.1093/aob/mcx154 29300872 PMC5906914

[B52] Louarn G. Faverjon L. Migault V. Escobar-Gutiérrez A. J. Combes D. (2016). “Assessment of ‘3DS’, a soil module for individual-based models of plant communities,” in IEEE International Conference on Functional-Structural Plant Growth Modeling, Simulation, Visualization and Applications (FSPMA). (Piscataway, N.J: IEEE ), 125–132. doi: 10.1109/fspma.2016.7818298

[B53] Lüscher A. Connolly J. Jacquard P. (1992). Neighbour specificity between Lolium perenne and Trifolium repens from a natural pasture. Oecologia 91, 404–409. doi: 10.1007/bf00317630 28313549

[B54] Lüscher A. Mueller-Harvey I. Soussana J. F. Rees R. M. Peyraud J. L. (2014). Potential of legume-based grassland–livestock systems in Europe: a review. Grass Forage Sci. 69, 206–228. doi: 10.1111/gfs.12124 26300574 PMC4540161

[B55] Maamouri A. Louarn G. Béguier V. Julier B. (2017). Performance of lucerne genotypes for biomass production and nitrogen content differs in monoculture and in mixture with grasses and is partly predicted from traits recorded on isolated plants. Crop Pasture Sci. 68, 942–951. doi: 10.1071/cp17052

[B56] Meilhac J. Durand J. L. Beguier V. Litrico I. (2019). Increasing the benefits of species diversity in multispecies temporary grasslands by increasing within-species diversity. Ann. Bot. 123, 891–900. doi: 10.1093/aob/mcy227 30615049 PMC6526319

[B57] Montazeaud G. Violle C. Roumet P. Rocher A. Ecarnot M. Compan F.. (2020). Multifaceted functional diversity for multifaceted crop yield: Towards ecological assembly rules for varietal mixtures. J. Appl. Ecol. 57, 2285–2295. doi: 10.1111/1365-2664.13735

[B58] Monteith J. L. (1977). Climate and the efficiency of crop production in Britain. Philos. Trans. R. Soc. London B Biol. Sci. 281, 277–294.

[B59] Moutier N. Baranger A. Fall S. Hanocq E. Marget P. Floriot M.. (2022). Mixing ability of intercropped wheat varieties: stability across environments and tester legume species. Front. Plant Sci. 13. doi: 10.3389/fpls.2022.877791 PMC9218859 35755684

[B60] Nyfeler D. Huguenin-Elie O. Suter M. Frossard E. Connolly J. Lüscher A. (2009). Strong mixture effects among four species in fertilized agricultural grassland led to persistent and consistent transgressive overyielding. J. Appl. Ecol. 46, 683–691. doi: 10.1111/j.1365-2664.2009.01653.x

[B61] Pagès L. Bécel C. Boukcim H. Moreau D. Nguyen C. Voisin A. S. (2014). Calibration and evaluation of ArchiSimple, a simple model of root system architecture. Ecol. Model. 290, 76–84. doi: 10.1016/j.ecolmodel.2013.11.014

[B62] Picheny V. Casadebaig P. Trépos R. Faivre R. Da Silva D. Vincourt P.. (2017). Using numerical plant models and phenotypic correlation space to design achievable ideotypes. Plant Cell Environ. 40, 1926–1939. doi: 10.1111/pce.13001 28626887

[B63] Pontes L. D. S. Maire V. Louault F. Soussana J. F. Carrère P. (2012). Impacts of species interactions on grass community productivity under contrasting management regimes. Oecologia 168, 761–771. doi: 10.1007/s00442-011-2129-3 21935663

[B64] Prieto I. Violle C. Barre P. Durand J. L. Ghesquiere M. Litrico I. (2015). Complementary effects of species and genetic diversity on productivity and stability of sown grasslands. Nat. Plants 1, 1–5. doi: 10.1038/nplants.2015.33 27247033

[B65] Prusinkiewicz P. Lindenmayer A. (1990). The Algorithmic Beauty of Plants (New York: Springer-Verlag).

[B66] R Core Team. (2022). R: a language and environment for statistical computing; (Vienna, Austria: R Core team), 2022. Available at: www.r-project.org (Accessed February 17, 2022).

[B67] Reiss E. R. Drinkwater L. E. (2018). Cultivar mixtures: a meta-analysis of the effect of intraspecific diversity on crop yield. Ecol. Appl. 28, 62–77. doi: 10.1002/eap.1629 28940830

[B68] Sinoquet H. Bonhomme R. (1992). Modeling radiative transfer in mixed and row intercropping systems. Agric. For. Meteorol. 62, 219–240. doi: 10.1016/0168-1923(92)90016-w

[B69] Smith S. E. (2004). “Breeding synthetic cultivars,” in Encyclopedia of plant and crop science. (New Brunswick, NJ: Rutgers University), 205–206.

[B70] Snyder L. D. Gómez M. I. Power A. G. (2020). Crop varietal mixtures as a strategy to support insect pest control, yield, economic, and nutritional services. Front. Sustain. Food Syst. 4, 60. doi: 10.3389/fsufs.2020.00060

[B71] Stomph T. Dordas C. Baranger A. de Rijk J. Dong B. Evers J.. (2020). Designing intercrops for high yield, yield stability and efficient use of resources: Are there principles? Adv. Agron. 160, 1–50. doi: 10.1016/bs.agron.2019.10.002

[B72] Thilakarathna M. S. Papadopoulos Y. A. Rodd A. V. Grimmett M. Fillmore S. A. E. Crouse M.. (2016). Nitrogen fixation and transfer of red clover genotypes under legume–grass forage based production systems. Nutrient Cycling Agroecosystems 106, 233–247. doi: 10.1007/s10705-016-9802-1

[B73] Vandermeer J. H. (1989). The Ecology of Intercropping (Cambridge: Cambridge University Press).

[B74] Vellend M. (2006). The consequences of genetic diversity in competitive communities. Ecology 87, 304–311. doi: 10.1890/05-0173 16637355

[B75] Verwimp C. Ruttink T. Muylle H. Van Glabeke S. Cnops G. Quataert P.. (2018). Temporal changes in genetic diversity and forage yield of perennial ryegrass in monoculture and in combination with red clover in swards. PloS One 13, e0206571. doi: 10.1371/journal.pone.0206571 30408053 PMC6224058

[B76] Viguier L. Bedoussac L. Journet E.-P. Justes E. (2018). Yield gap analysis extended to marketable grain reveals the profitability of organic lentil-spring wheat intercrops. Agron. Sustain. Dev. 38 (4), 39. doi: 10.1007/s13593-018-0515-5

[B77] Violle C. Enquist B. J. McGill B. J. Jiang L. I. N. Albert C. H. Hulshof C.. (2012). The return of the variance: intraspecific variability in community ecology. Trends Ecol. Evol. 27, 244–252. doi: 10.1016/j.tree.2011.11.014 22244797

[B78] Weigelt A. Jolliffe P. (2003). Indices of plant competition. J. Ecol. 91 (5), 707–720. doi: 10.1046/j.1365-2745.2003.00805.x

[B79] Weiner J. Solbrig O. T. (1984). The meaning and measurement of size hierarchies in plant populations. Oecologia 61, 334–336. doi: 10.1007/bf00379630 28311058

[B80] Westerband A. C. Funk J. L. Barton K. E. (2021). Intraspecific trait variation in plants: a renewed focus on its role in ecological processes. Ann. Bot. 127, 397–410. doi: 10.1093/aob/mcab011 33507251 PMC7988520

[B81] Whitlock R. (2014). Relationships between adaptive and neutral genetic diversity and ecological structure and functioning: a meta-analysis. J. Ecol. 102, 857–872. doi: 10.1111/1365-2745.12240 25210204 PMC4142011

[B82] Williams T. A. Abberton M. T. Rhodes I. (2003). Performance of white clover varieties combined in blends and alone when grown with perennial ryegrass under sheep and cattle grazing. Grass Forage Sci. 58, 90–93. doi: 10.1046/j.1365-2494.2003.00349.x

[B83] Wolfe M. S. (1985). The current status and prospects of multiline cultivars and variety mixtures for disease resistance. Annu. Rev. Phytopathol. 23, 251–273. doi: 10.1146/annurev.phyto.23.1.251

[B84] Zannone L. Assemat L. Rotili P. Jacquard P. (1983). An experimental study of intraspecific competition within several forage crops (1). Agronomie 3, 451–459. doi: 10.1051/agro:19830508

[B85] Zhang B. DeAngelis D. L. (2020). An overview of agent-based models in plant biology and ecology. Ann. Bot. 126, 539–557. doi: 10.1093/aob/mcaa043 32173742 PMC7489105

