# Supplementary material for: Impact of intraspecific genetic variation on interspecific competition: a theoretical case study of forage binary mixtures
Source: Front Plant Sci. 2024 Sep 30;15:1356506. doi: 10.3389/fpls.2024.1356506 (PMC11482038; doi:10.3389/fpls.2024.1356506)
Supplement: Supplementary file 1 [file DataSheet1.docx]

**Supplementary materials**


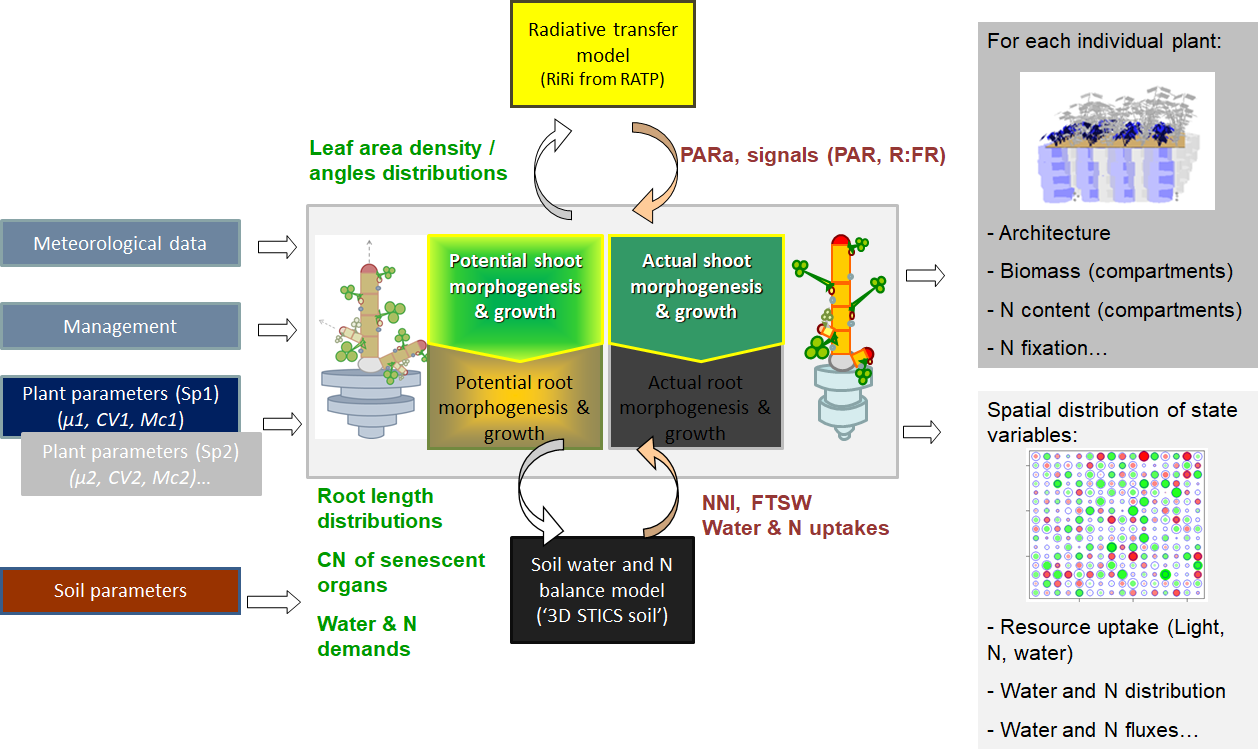


**Fig. Sup. A:** Diagrammatic representation of the Virtual Grassland (VGL) model, associating a plant model (L-egume, blue boxes) with environmental models of radiative transfer (yellow box) and soil functioning (brown box). Variables in green and brown represent the main exchange variables between sub-models. The major outputs of the VGL model are indicated in the grey boxes on the right. New features were introduced for describing within-species plant parameter variation (CV and Mc as new inputs).


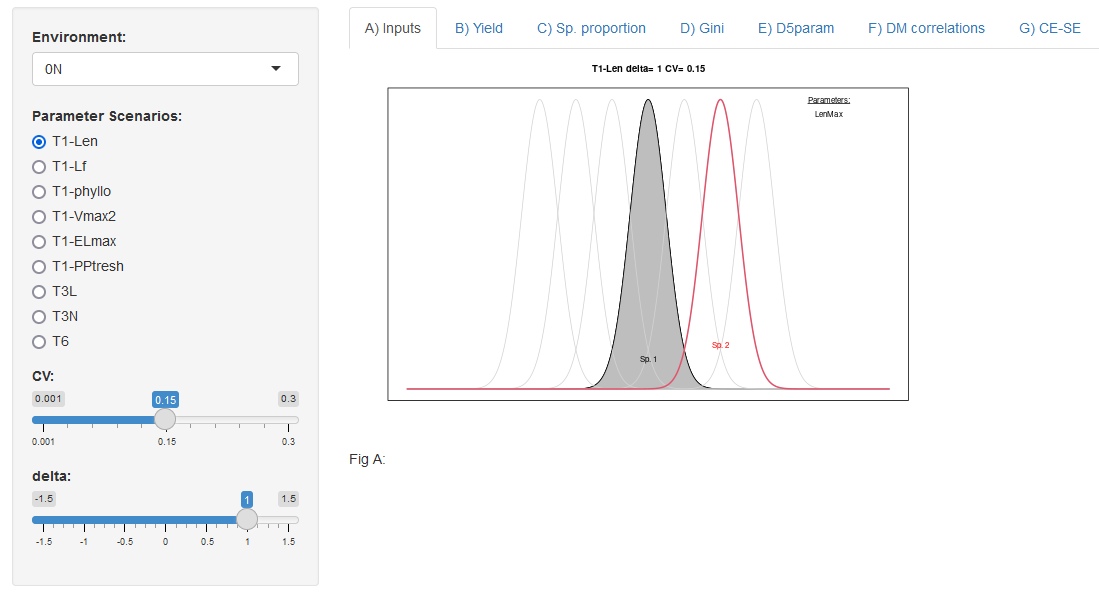


**Fig. Sup. B:** Graphical user interface of the online interactive app built to explore model outputs in the different simulated scenarios. Panel A) for exploring plant parameter distributions; panel B) for total annual yield and overyielding; panel C) for species proportion; panel D) for Gini coefficients in both species; panel E) for the shifts in D5_param_ in all parameters; panel F) for correlation coefficients between individual plant biomass and the various indices of plant performance and access to resources; panel G) for Loreau & Hector’s complementarity and selection effects.

Web link: <https://p3f-inrae.shinyapps.io/shiny_BLW_1/>

Github repository: <https://github.com/glouarn/ShinyApp-binder/tree/master/shiny-blw-article1>

**Fig. Sup. C.** Response of community stability (p50s, A-F) and total annual productivity (Ytot, G-L) to the gradient of mean trait divergence in T1 communities with low within-species variation (CV=0.001). Blue and brown boxes stand for simulations under 0N and N+ environments, respectively.

**Fig. Sup. D.** Response of community overyieding (OY) to the gradient of mean trait divergence in T1 communities with low within-species variation (CV=0.001). Blue and brown boxes stand for simulations under 0N and N+ environments, respectively.

**Fig. Sup. E.** Responses of community stability (p50s, A-F) and total annual productivity (Ytot, G-L) to the level of IV in situations corresponding to a null model of interspecific competition (Δ=0) in T1 communities under N+ and change in D5_param_ for the different parameters at high IV (CV=0.30).

**Fig. Sup. F.** Responses of community stability (p50s) to the gradient of mean trait divergence in T3L (AC), T3N (BD) and T6 (CE) communities with high within-species variation (CV=0.30). Blue and brown boxes indicate simulations under 0N and N+ environments, respectively. Grey dashed line: average species proportion at low within-species variation. Length of black vertical bars equal the stabilising effects of IV (S_IV_) compared to the reference simulation at low within-species variation. n=9 replicate simulations by community.

**Fig. Sup. G.** Correlations between plant biomass and the multi-parameter score (P_score_) of each plant for T3L (A), T3N (B) and T6 (C) communities at different levels of IV. Data in each community are for all the individuals of Sp. 2 under N+ environment.

**Fig. Sup. H.** Examples of shifts in D5_param_ for maximal internode length (ACEG) and maximal root elongation rate (BDFH) parameters at high IV (CV=0.30) for Sp2 in T3N (AB), T3L (CD) and T6 (EFJH) communities. Blue and brown boxes indicate simulations under 0N and N+ environments, respectively. Grey dashed line: theoretical value of D5_param_ in the absence of selection. n=9 replicate simulations by community.

**Fig. Sup. I.** Example of simulated individual plant biomass distributions within a community (A) and relationships between individual plant biomass and cumulated PAR intercepted (PARi, B) and cumulated mineral N uptake (Nupt, C) by the plant. Data are for a simulation of the community T3L/+0.5Δ. Blue and brown points indicate simulations under 0N and N+ environments, respectively.

**Sup. Table A.** Literature review for the within-species variation of different shoot and root parameters of the L-egume model. n: number of genotypes or cultivars in the study; SD: standard deviation; CV: coefficient of variation

| **Parameter** | **Unit** | **Description** | **Species** | **n** | **Mean** | **SD** | **CV** | **Reference** |
| --- | --- | --- | --- | --- | --- | --- | --- | --- |
| Phyllo1 | °Cd | Phyllochron on main shoot | *Medicago sativa* | 5 | 38.5 | 3.5 | 0.09 | Zaka et al., 2017 |
|  |  | axis | *Medicago sativa* | 5 | 35.8 | 2.04 | 0.06 | Gentelet, 2021 |
|  |  |  | *Festuca arundinacea* | 5 | 52.6 | 3.125 | 0.06 | Zaka et al., 2017 |
|  |  |  | *Sorghum bicolor* | 9 | 60.5 | 5.75 | 0.10 | Craufurd et al., 1998 |
|  |  |  |  |  |  |  |  |  |
| LmaxLf | cm | Maximal leaf length | *Medicago sativa* | 5 | 3.75 | 0.45 | 0.12 | Zaka et al., 2017 |
|  |  |  | *Medicago sativa* | 40 | 3.53 | 0.45 | 0.13 | Maamouri et al., 2017 |
|  |  |  | *Medicago sativa* | 5 | 3.51 | 0.29 | 0.08 | Gentelet, 2021 |
|  |  |  | *Festuca arundinacea* | 5 | 15 | 2.625 | 0.18 | Zaka et al., 2017 |
|  |  |  | *Trifolium pratense* | 5 | 5.31 | 0.86 | 0.16 | Gentelet, 2021 |
|  |  |  |  |  |  |  |  |  |
| LmaxIn | cm | Maximal Internode length | *Medicago sativa* | 40 | 3.3 | 0.775 | 0.23 | Maamouri et al., 2017 |
|  |  |  | *Medicago sativa* | 5 | 10.33 | 1.11 | 0.11 | Gentelet, 2021 |
|  |  |  |  |  |  |  |  |  |
| NBsh | - | Maximal number of | *Medicago sativa* | 40 | 59 | 18 | 0.31 | Maamouri et al., 2017 |
|  |  | primary shoots | *Trifolium pratense* | 5 | 6.24 | 1.96 | 0.31 | Gentelet, 2021 |
|  |  |  |  |  |  |  |  |  |
| RUEmax | kg.MJ-1 | Maximal Radiation Use | *Triticum aestivum* | NA | 0.0378 | 0.001 | 0.03 | Slattery & Ort, 2015 |
|  |  | Efficiency | *Zea mays* | NA | 0.0488 | 0.001 | 0.02 | Slattery & Ort, 2015 |
|  |  |  | *Sorghum bicolor* | NA | 0.0455 | 0.002 | 0.04 | Slattery & Ort, 2015 |
|  |  |  | *Glycine max* | NA | 0.0282 | 0.001 | 0.04 | Slattery & Ort, 2015 |
|  |  |  | *Cajanum arietinum* | NA | 0.0283 | 0.002 | 0.07 | Slattery & Ort, 2015 |
|  |  |  |  |  |  |  |  |  |
| IBD | mm | Average root inter-branch | *Medicago sativa* | 5 | 0.54 | 0.1 | 0.19 | Gentelet, 2021 |
|  |  | distance | *Solanum lycopersicum* | 10 | 2.5 | 0.259 | 0.10 | Bui et al., 2015 |
|  |  |  | *Solanum melongena* | 9 | 3.12 | 0.54 | 0.17 | Bui et al., 2015 |
|  |  |  | *Capsicum annuum* | 7 | 4.06 | 0.58 | 0.14 | Bui et al., 2015 |
|  |  |  | *Brassica napus* | 8 | 4.2 | 0.6 | 0.14 | Lecarpentier et al., 2021 |
|  |  |  |  |  |  |  |  |  |
| Dmax | mm | Maximum root apex | *Medicago sativa* | 5 | 0.15 | 0.01 | 0.07 | Gentelet, 2021 |
|  |  | diameter | *Solanum lycopersicum* | 10 | 0.752 | 0.129 | 0.17 | Bui et al., 2015 |
|  |  |  | *Solanum melongena* | 9 | 0.753 | 0.097 | 0.13 | Bui et al., 2015 |
|  |  |  | *Capsicum annuum* | 7 | 0.943 | 0.111 | 0.12 | Bui et al., 2015 |
|  |  |  | *Brassica napus* | 8 | 0.68 | 0.055 | 0.08 | Lecarpentier et al., 2021 |
|  |  |  |  |  |  |  |  |  |
| ELmax | mm.°Cd-1 | Elongation rate of roots | *Medicago sativa* | 5 | 0.03 | 0.01 | 0.33 | Gentelet, 2021 |
|  |  | at the maximal apex | *Solanum lycopersicum* | 10 | 2.47 | 0.468 | 0.19 | Bui et al., 2015 |
|  |  | diameter | *Solanum melongena* | 9 | 1.77 | 0.228 | 0.13 | Bui et al., 2015 |
|  |  |  | *Capsicum annuum* | 7 | 1.81 | 0.35 | 0.19 | Bui et al., 2015 |
|  |  |  |  |  |  |  |  |  |
| DlDm | - | Slope of the relationship | *Medicago sativa* | 5 | 0.61 | 0.0525 | 0.09 | Gentelet, 2021 |
|  |  | between parent and | *Solanum lycopersicum* | 10 | 0.268 | 0.0417 | 0.16 | Bui et al., 2015 |
|  |  | lateral root diameters | *Solanum melongena* | 9 | 0.281 | 0.0581 | 0.21 | Bui et al., 2015 |
|  |  |  | *Capsicum annuum* | 7 | 0.327 | 0.0212 | 0.06 | Bui et al., 2015 |
|  |  |  | *Brassica napus* | 8 | 0.33 | 0.04 | 0.12 | Lecarpentier et al., 2021 |
|  |  |  |  |  |  |  |  |  |
| Vmax2 | mol.m-1.°Cd-1 | Maximum absorption rate of LATS | *Triticum aestivum* | 3 | 0.05 | 0.01 | 0.20 | Ruget et al., 2002 |
|  |  |  |  |  |  |  |  |  |
| PPtreshH | h | Treshold photoperiod | *Medicago sativa* | 3 | 15 | 1.5 | 0.10 | Ta et al., 2020 |
|  |  | response |  |  |  |  |  |  |

**Sup Table B.** Mean parameter values used for Sp2 in the range of T6 combinations tested. Parameters for Sp1 were fixed and correspond to the 0 Δ.

|  | -1,5 Δ | -1 Δ | -0,5 Δ | 0 Δ | +0,5 Δ | +1 Δ | +1,5 Δ |
| --- | --- | --- | --- | --- | --- | --- | --- |
| LmaxIn | 2,1175 | 2,6950 | 3,2425 | 3,8500 | 4,4275 | 5,0050 | 5,5825 |
| LmaxL | 1,3750 | 1,7500 | 2,1250 | 2,5000 | 2,8750 | 3,2500 | 3,6250 |
| Vmax2 | 0,0275 | 0,0350 | 0,0425 | 0,0500 | 0,0575 | 0,0650 | 0,0725 |
| ELmaxR | 0,0561 | 0,0714 | 0,0867 | 0,1020 | 0,1173 | 0,1326 | 0,1479 |
| Phyllo1 | 50,750 | 45,500 | 40,250 | 35,000 | 29,750 | 24,500 | 19,250 |
| PPtreshh | 13,775 | 12,350 | 10,925 | 9,500 | 8,075 | 6,650 | 5,225 |

**Supplementary Table C**: Detailed simulation protocol for running the virtual experiment.

| *Definition of the 63 plant communities* | - A series of 63 virtual binary mixtures was generated combining a reference species (hereafter called Sp1 for species 1, corresponding to the G- morphotype described in Louarn and Faverjon, 2018) and a second species (Sp2) defined by modifying the values of a series of one to six parameters compared to Sp1 (Figure 1).  - For the boundaries of within-species variation, a maximal standard deviation for each plant parameter (*σ_max_*) was defined according to the mean parameter values of the reference calibration (G- morphotype, Louarn and Faverjon, 2018) and the highest possible level of CV recorded in the literature review reported in Supplementary Table A (*σ_max_* = µSp1*0.3).  - For between-species variability, we chose the between species difference in a mixture as a multiple *σ_max_*. Seven possible levels of mean trait divergence (Δ) were defined for each parameter, ranging from -1.5**σ_max_* to +1.5**σ_max_* (Figure 1; Fig. Sup B-a). The median case of 0 *σ_max_* (Δ=0) represented a particular case, which is not properly an intercrop (more a mixture of two varieties from the same species, as mean trait values were identical), but which served as a useful reference (i.e. null model) to disentangle the effects mean trait divergence and IV on model outputs.  - Six parameters were selected in this study to build plant communities with contrasting levels of competition between species. They were identified from a sensitivity analysis previously published on the model using the Sp1 parametrisation (Louarn et al., 2020). They consisted in three parameters identified as having a marked influence on light competition (namely L_max_L, L_max_In and Phyllo_1_ standing for the maximal leaf length, maximal internode length and maximal phyllochron of primary shoot axes, respectively) and three parameters with a marked influence on soil mineral N competition (namely Vmax_2_, EL_max_R and PPtresh_H_ standing for the maximum rate of absorption achieved by LATS transporters, the elongation rate of roots at maximal apex diameter and the photoperiod threshold required to start inducing a reduction in plant development, respectively). All parameter differences did not act in the same direction to influence the competition between species (i.e. some increasing parameter values have a positive effect on competitive ability, while others have a negative effect). In order to keep a simple interpretation of model outputs in terms of resource partitioning between species with a limited simulation design, two conventions were defined to select among the possible binary mixture tested:  * The first was to apply increasing Δ for each parameter according to one species that was favoured in the acquisition of a given resource,  * The second was to differentiate the competitive ability of Sp1 and Sp2 for the two contested resources.  As a consequence, the gradients of increasing Δ values were defined to favour the competitive ability of Sp1 with respect to Sp2 for the three parameters chosen for their effect on light competition (L_max_L, L_max_In and Phyllo_1_). Similarly, the gradients of increasing delta values was defined to favour Sp2 over Sp1 for the three parameters directly affecting N competition (Vmax_2_, EL_max_R and PPtresh_H_).  - Finally, the community tested could also differ in terms of the number of parameters (or ‘traits’) for which the two species differed (Figure 1). Communities with species differing by 1, 3 or 6 trait values were tested (T1, T3 and T6). Not all possible combinations were included for T3. Non-random combinations of traits were chosen to produce regular gradients of competitive advantage for either light or N acquisition with increasing Δ values. Thus, for T3, two situations accounting for the three light competition parameters (T3L) and the three N competition parameters (T3N) grouped together were selected. This resulted in nine possible trait combinations that defined a range of 63 virtual plant communities (i.e. (6*T1 + T3L +T3N +T6) * 7Δ). These scenarios included a series of nine situations at Δ equals zero (i.e. no trait divergence between the mixed species) that corresponded to null models of interspecific competition for each of the T1 to T6 treatments. In these controls, the expected outcome of the competition between the two species was theoretically known (i.e. total yield equals the yield of a pure Sp1 stand; species proportion remains equal to sowing proportions).  - Supplementary Table B summarises the different parameter values achieved by Sp2 under the different scenarios tested. |
| --- | --- |
| *Levels of IV studied* | - In order to assess the impact of within-population parameter variation on interspecific competition, simulations with all 63 plant mixtures were run by testing the impact of the three possible levels of IV: a very low (CV=0.001), moderate (CV=0.15) and high (CV=0.30) level of intraspecific variation (Figure 1). In each community, CV inputs were only applied to the parameter involved in the mean trait divergence between species, with the same CV value applied to all parameters. |
| *Initial conditions* | - The simulations were run assuming dense stands of plants (16x16 = 256 plants at 400 plants m^-2^) with a regular spacing and distribution of species according to a checkerboard arrangement.  - The plot location was typical of Western European temperate grasslands (Lusignan, France, 46.26°N, 0.11°E). The edaphic conditions at this site corresponded to a Dystric Cambisol (IUSS Working Group WRB 2006) with silty-loamy texture in the surface and clay in the subsoil horizons. A soil calibration available for this site was used, which corresponded to a 1.5-meter deep soil with 268 mm available water at field capacity, and 1.1 g organic N.kg-1 in the upper soil layer. Under low to moderate N fertilisation, this soil generally produces N-limiting growing conditions for non-fixing grassland species, even when grown in mixtures (Louarn et al., 2015).  - The date of emergence was assumed to occur on March 1st (DOY 61) in average. The initial variability of the germination date were set at the same value for all species (standard deviation of 3 degree.days).  -The first year of production after stand establishment was simulated and analysed during this study. |
| *Environmental conditions* | - We considered 30-year daily average weather data (1986- 2016) at the plot location as the meteorological inputs for the simulation.  - The RiRi turbid medium model from RATP is used in VGL to compute light interception by individual plants (Sinoquet et al., 2001; Figure Sup. A). This model requires daily meteorological data, and a discretized representation of leaf area density distribution and leaf angle distribution into a 3D grid for each plant to be run. As in Louarn and Faverjon (2018), a voxel size of 4x4x2 cm and a sky discretization following a Den-dusk’s TURTLE with 6 directions were used  - The 3DS soil model is used in VGL to compute daily water and mineral N uptakes by individual plants (Louarn et al., 2016; Figure Sup. A). As for light, a 3D grid with voxels of 4x4x2 cm was used.  - A toricity of the 3D grids was assumed above- and below-ground to avoid border effects.  - Two different series of simulations were run: a first soil environment was designed to favour high N competition and was run under low soil N availability (0N, no fertilisation). A second condition was designed to ensure high soil N availability and generate high light competition (N+, mineral N fertilisation set at 400 kg N ha^-1^). |
| *Management* | - In order to avoid competition for water, an irrigation calendar that matched potential evapotranspiration and crop water requirements was applied. At total of 330 mm was applied during the 1-year simulation.  - Four harvests were carried out during the year according to a fixed calendar (days 189, 230, 283 and 335 of the year). |
| *Replicates* | - The simulations were repeated nine times for each combination of plant community, within-species diversity and environment (N+/0N). A total of approximately 7000 independent simulations were performed for this study. |

**Supplementary Table D**: Total biomass production of the pure species (Ypur, in g.m^-2^) simulated under high (N+) and low (0N) soil fertility (CV=0.001). Simulation ID’s correspond to the numbers used to identify each species in the published dataset.
